# Monitoring complete hydatidiform molar pregnancies after normalisation of human chorionic gonadotrophin: national retrospective population study

**DOI:** 10.1136/bmjmed-2024-001017

**Published:** 2025-04-23

**Authors:** Brenna E Swift, Leonoor Coopmans, Kam Singh, Christopher Coyle, Edmund H Wilkes, Imran Jabbar, Geoffrey Maher, Xianne Aguiar, Lina Salman, Julia E Palmer, Naveed Sarwar, Reece Caldwell, Eshan Ghorani, Baljeet Kaur, Nienke E van Trommel, Christianne A R Lok, Matthew Winter, Michael J Seckl

**Affiliations:** 1 Department of Obstetrics and Gynaecology, University of Toronto, Toronto, Ontario, Canada; 2 Department of Gynaecologic Oncology, Netherlands Cancer Institute-Antoni van Leeuwenhoek Hospital, Amsterdam, Netherlands; 3 Sheffield Trophoblastic Disease Centre, Sheffield Teaching Hospitals NHS Foundation Trust, Sheffield, UK; 4 Department of Medical Oncology, Portsmouth Hospitals University NHS Trust, Portsmouth, UK; 5 Department of Clinical Biochemistry, NHS North West London Pathology, London, UK; 6 Department of Clinical Chemistry, Sheffield Teaching Hospitals NHS Foundation Trust, Sheffield, UK; 7 Charing Cross Gestational Trophoblastic Centre, Imperial College London-Charing Cross Campus, London, UK; 8 Charing Cross Gestational Trophoblastic Centre, Department of Medical Oncology, Imperial College London-Charing Cross Campus, London, UK; 9 Division of Gynaecologic Oncology, London Health Sciences Centre, University of Western Ontario, London, Ontario, Canada; 10 Sheffield Trophoblastic Disease Centre, Department of Gynecologic Oncology, Sheffield Teaching Hospitals NHS Foundation Trust, Sheffield, UK; 11 Gestational Trophoblastic Centre, Department of Medical Oncology, Imperial College London-Charing Cross Campus, London, UK; 12 Department of Pathology, Gestational Trophoblastic Centre, Imperial College London-Charing Cross Campus, London, UK; 13 Sheffield Trophoblastic Disease Centre, Weston Park Cancer Centre, Department of Medical Oncology, Sheffield Teaching Hospitals NHS Foundation Trust, Sheffield, UK; 14 Charing Cross Gestational Trophoblastic Centre, Imperial College London-Hammersmith Hospitals Campus, London, UK

**Keywords:** Medical oncology

## Abstract

**Objective:**

To provide evidence for a reduced surveillance protocol to detect gestational trophoblastic neoplasia after normalisation of human chorionic gonadotrophin (hCG) levels following uterine evacuation of a complete hydatidiform mole.

**Design:**

National retrospective population study

**Setting:**

Two UK Trophoblastic Disease Treatment Centres (Sheffield and London), 1 January 1980 to 30 November 2020.

**Participants:**

17 424 patients with hCG normalisation after evacuation of their complete hydatidiform mole were included. Complete hydatidiform moles were verified by centralised pathological review. Patients were excluded if lost to follow-up or required treatment before normalisation of hCG levels.

**Main outcome measures:**

Incidence and clinical presentation of gestational trophoblastic neoplasia after normalisation of hCG levels following uterine evacuation of a complete hydatidiform mole.

**Results:**

Of 17 424 patients whose hCG normalised after complete hydatidiform mole evacuation, 99.8% (n=17 393 of 17 424) did not subsequently develop gestational trophoblastic neoplasia. The overall risk of gestational trophoblastic neoplasia after previous normalisation of hCG levels was 0.2% (n=31 of 17 424 patients). The risk of developing gestational trophoblastic neoplasia after uterine evacuation was substantially lower if hCG levels returned to normal in <56 days rather than ≥56 days (posterior medians 0.06%, 95% credible interval 0.01% to 0.14% *v* 0.22%, 0.15% to 0.31%), with a posterior relative risk of 0.25 (0.06 to 0.72). Most patients who developed gestational trophoblastic neoplasia (71.0%, n=22 of 31) had received a diagnosis after the current six month surveillance protocol. The cumulative risk of developing gestational trophoblastic neoplasia in patients whose hCG levels normalised early increased minimally with time. If a patient had normal hCG levels in <56 days, a clinically relevant time point, the risk of developing gestational trophoblastic neoplasia was small (0.04%, about 1 in 2619 patients) at 39 months after normalisation. The equivalent risk for a patient who had normal hCG levels in ≥56 days was 0.16% (about 1 in 642 patients). All 31 women who developed gestational trophoblastic neoplasia achieved sustained remission after subsequent treatment.

**Conclusions:**

The findings of the study indicate that surveillance protocols could safely change to one confirmatory normal hCG value for patients whose hCG levels return to normal in <56 days of evacuation of a complete hydatidiform mole. Patients whose hCG levels return to normal in ≥56 days should be counselled on the remaining risk of gestational trophoblastic neoplasia over time to help decide the length of subsequent follow-up.

**WHAT IS ALREADY KNOWN ON THIS TOPIC**

- In patients who have had uterine evacuation of a partial hydatidiform mole, the risk of gestational trophoblastic neoplasia after levels of human chorionic gonadotropin (hCG) have returned to normal is low, therefore only one confirmatory normal hCG value is recommended before attempting a new pregnancy
- In contrast, in patients with a complete hydatidiform molar pregnancy, who have a higher risk of gestational trophoblastic neoplasia after uterine evacuation, monitoring is longer, even if hCG levels return to normal in <56 days

**WHAT THIS STUDY ADDS**

- Relative risks are reported of malignant transformation to gestational trophoblastic neoplasia over time when hCG levels have normalised in <56 or ≥56 days in patients with a complete hydatidiform mole
- The findings indicate that monitoring of hCG levels for complete hydatidiform mole can be safely stopped after one confirmatory normal hCG value if normalisation occurred in <56 days

**HOW THIS STUDY MIGHT AFFECT RESEARCH, PRACTICE, OR POLICY**

- Data from this study could contribute to an informed discussion between healthcare workers and patients if hCG levels normalise in ≥56 days
- The findings of this study will reduce anxiety and enable many more women to start their next pregnancy earlier

## Introduction

 Complete and partial hydatidiform moles are abnormal conceptions affecting 1800 women each year in the UK (about 220 000 women globally a year) or about 1-3 per 1000 pregnancies.^1^ A complete hydatidiform mole is an androgenetic diploid conception lacking maternal nuclear DNA but with two paternal sets of chromosomes. A partial hydatidiform mole is a triploid pregnancy with one maternal and two paternal chromosomal contributions. Molar pregnancies result in aggressive abnormal placental (trophoblast) development that typically causes bleeding in the first trimester of pregnancy. Some molar pregnancies might be undetected because of early spontaneous abortions, but when an abnormal pregnancy suggestive of a molar pregnancy is detected on an early pelvic ultrasound, suction uterine evacuation is usually performed. Histological examination of the evacuated material is essential to make a diagnosis of complete hydatidiform mole, partial hydatidiform mole, or non-molar pregnancy loss. All patients with a histologically confirmed complete or partial hydatidiform mole should then undergo serial measurements of serum human chorionic gonadotropin (hCG) levels to ensure no regrowth of residual trophoblastic tissue has occurred.^2^ Persistence of trophoblastic tissue occurs in about 15% of complete hydatidiform moles and 0.5–1% of partial hydatidiform moles where levels of hCG might increase or plateau over at least two or three consecutive values, respectively, indicating the onset of malignant change, also known as postmolar gestational trophoblastic neoplasia.^3 4^ Early detection is important so that subsequent treatment can achieve a nearly complete cure rate. Almost all postmolar gestational trophoblastic neoplasias will occur before serum levels of hCG have normalised after uterine evacuation, but in a few cases, malignancy can occur after the first normal hormone value.^5^

We have previously shown that the risk of gestational trophoblastic neoplasia after hCG levels return to normal in patients with a partial hydatidiform mole is low (0.03%, n=3 of 9586 patients) and therefore, we recommended one confirmatory normal value before stopping hCG monitoring.^6^ In contrast, for a complete hydatidiform mole, we noted a slightly higher risk of postmolar gestational trophoblastic neoplasia after hCG levels had returned to normal (0.24%, n=20 of 8400 patients) which decreased from one in 839 (0.12%) patients at four months to one in 1677 (0.06%) patients at 12 months after the first normal hCG value.^6^ In keeping with previous data,^7 8^ patients with a complete hydatidiform mole who achieved normalisation of hCG levels in <56 days had a significantly lower risk of gestational trophoblastic neoplasia than those with normal levels in ≥56 days (one in 1159 (0.09%) *v* one in 308 (0.32%) patients, odds ratio 0.27, 95% confidence interval 0.08 to 0.88, P=0.03). Consequently, patients with a complete hydatidiform mole seemed to need longer monitoring if hCG levels returned to normal in ≥56 days from the date of uterine evacuation. In the UK, hCG monitoring is continued for six more months after the first normal hCG value in these patients, whereas those with normal hCG levels in <56 days are monitored for only six months from the date of molar evacuation.^7^ Globally, many other centres monitor patients with a complete hydatidiform mole for several months after a normal hCG level.^9^

Prolonged monitoring after normalisation of hCG levels after uterine evacuation of a complete hydatidiform mole can cause considerable distress for patients and delay their ability to attempt another pregnancy. Patients report disruption of their wellbeing by feelings of anxiousness (47%), depression (27%), and distress (70%) because of receiving a diagnosis of gestational trophoblastic disease.^10^ Hence, we thought it might be possible to shorten the duration of hCG monitoring when hCG levels had returned to normal after a complete hydatidiform mole, particularly in those who achieved their first normal value in <56 days. Here, in a larger cohort of patients from two trophoblastic disease centres, we reviewed the relative risk of malignant transformation over time in patients with a complete hydatidiform mole who had normal hCG levels in <56 and ≥56 days.

## Methods

All registered patients with a complete hydatidiform mole verified by centralised histopathological review in Charing Cross Hospital and Sheffield Trophoblastic Disease Centres were identified from the respective databases between 1 January 1980 and 30 November 2020 to ensure at least two years of follow-up. Patients were excluded if they had received treatment for gestational trophoblastic neoplasia before normalisation of hCG levels, had a twin pregnancy with a complete hydatidiform mole and a healthy co-twin, had a recurrent mole, or if follow-up of hCG levels were incomplete. Patients with histologically unclassified molar pregnancies were included (12.6% of the total studied population) because our previous analysis showed that these patients behaved like patients with a complete hydatidiform mole in terms of their risk of postmolar gestational trophoblastic neoplasia.^6^

Serum and urine levels of hCG were measured with the Charing Cross radioimmunoassay and Siemens Immulite assay, as previously described.^2^ Samples were taken once every two weeks until hCG levels were normal and then monthly until six months after evacuation or for another six months if normalisation of hCG levels occurred in ≥56 days after the evacuation. Older surveillance protocols (ie, before 2000) during the study period included up to two years of monthly hCG samples.^11^

Time to normalisation of hCG level was calculated from the date of uterine evacuation until the first normal serum hCG value. Gestational trophoblastic neoplasia was diagnosed according to the criteria of the International Federation of Gynaecology and Obstetrics (FIGO) for postmolar gestational trophoblastic neoplasia. Time to diagnosis of gestational trophoblastic neoplasia was determined from the date of the first normal hCG value to the start of treatment for gestational trophoblastic neoplasia, which in the UK is the same day as diagnosis. The relative risk of developing gestational trophoblastic neoplasia was calculated over time with regard to normalisation of hCG levels in <56 or ≥56 days.

For patients who developed gestational trophoblastic neoplasia, clinical presentation and interval pregnancy history were recorded, as well as FIGO score and treatment required. If biopsy material from the gestational trophoblastic neoplasia was available, microsatellite polymorphism analysis was undertaken to assess whether the preceding molar pregnancy was causative of the subsequent tumour, as previously described.^12^

Two bayesian models were fitted to the data presented here with the brms R package.^13 14^ Firstly, a model was fitted to examine the overall incidences of gestational trophoblastic neoplasia in each patient group in order to compare differences between them across the entire period studied. This first model was fitted as specified in equations 1-3, where *y_i_* represents the number of events in group *i*; n*_i_* and p*_i_* represent the number of patients and probability of developing gestational trophoblastic neoplasia in group *i* represent the number of patients and probability of developing gestational trophoblastic neoplasia in group *i*, respectively; and α*_i_* represents the coefficient for group *i*. A relatively informative prior distribution for the probability of the development of gestational trophoblastic neoplasia was set at a suitably low level as, a priori, we knew from previous data that the risk of developing gestational trophoblastic neoplasia is low.^6^ We did, however, have a relatively wide variance on the prior distribution to reflect our uncertainty before seeing the data (equation 3). Secondly, a bayesian time-to-event (ie, Kaplan-Meier equivalent) model was fitted to the data. This model was specified as described in equations 4-6 below, where H (t*_ij_*) represents the risk of developing gestational trophoblastic neoplasia at time interval *i*, in patient group *j*; n*_ij_* represents the number of patients at risk in time interval *i*, in patient group *j*; p*_ij_* represents the probability of developing gestational trophoblastic neoplasia in time interval *i*, in patient group *j*; and α*_ij_* represents the coefficient for time interval *i*, in patient group *j*. The prior distribution for the risk of developing gestational trophoblastic neoplasia in each time window was assumed, a priori, to be lower (equation 6) than the overall risk of developing gestational trophoblastic neoplasia over the entire time period (equation 3). Posterior distributions are summarised as the 2.5th, 50th, and 97.5th centiles in each case. All Markov Monte Carlo chains converged for each of the parameters in the model (*R*=1.0). The online supplemental material has the R code required to reproduce these analyses. Online supplemental figures 2–4 have more detailed information on a comparison of the statistical models used here and an analysis of the influence of the chosen prior distributions. The analysis of the influence of the chosen prior distributions shows how, while the prior regularises the model’s inferences, the data have a strong influence on the posterior.


(1) $$y_{i}∼Binomial\left(n_{i},p_{i}\right)$$

(2) $$logit\left(p_{ij}\right)=\alpha_{i}Group_{j}$$

(3) $$\alpha_{i}∼N\left(mean=-8,SD=3\right)$$

(4) $$H\left(t_{ij}\right)∼Binomial\left(n_{ij},p_{ij}\right)$$

(5) $${logit(p}_{ij})=\alpha_{ij}\left({Interval}_{i}\times {Group}_{j}\right)$$

(6) $$\alpha_{ij}∼N\left(-12,\;3\right)$$

### Patient and public involvement

Patients and/or the public were not involved in the design, or conduct, or reporting, or dissemination plans of this research. This study was approved as an NHS service evaluation by Imperial College NHS Healthcare Trust and Sheffield Teaching Hospitals Trust and ethics review was therefore not required. Previous patients will not be contacted about the service evaluation. The knowledge gained from this service evaluation has influenced new treatment protocols that will be communicated to new patients in the clinic and to other providers through conference presentations, research publication and clinical practice guidelines.

## Results

**Figure 1:**
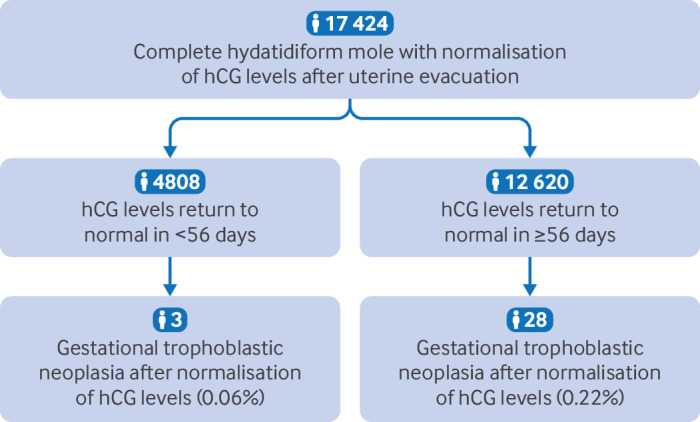
Patients who achieved normal serum levels of human chorionic gonadotropin (hCG) in <56 and ≥56 days after uterine evacuation of a complete hydatidiform mole and those who subsequently developed gestational trophoblastic neoplasia

Between 1980 and 2020, 17 424 patients achieved normal serum levels of hCG after uterine evacuation of a complete hydatidiform mole, and 99.8% (n=17 393 of 17 424) did not subsequently develop gestational trophoblastic neoplasia (figure 1). The overall risk of gestational trophoblastic neoplasia after previous normalisation of hCG levels was 0.2% (n=31 of 17 424 patients). All 31 women treated for gestational trophoblastic neoplasia with systemic chemotherapy had remission of disease. One patient with high risk disease, however, who had received one cycle of low dose etoposide and cisplatin followed by five cycles of etoposide, methotrexate, and dactinomycin, alternating weekly with cyclophosphamide and vincristine, subsequently developed a second malignancy and died of acute leukaemia.

Time from normalisation of hCG levels to the development of gestational trophoblastic neoplasia in the 31 patients who developed the neoplasia after normalisation of hCG levels ranged from 0.13 months to 89.9 months. Most patients (71.0%, n=22 of 31) received a diagnosis after the existing hCG surveillance protocol.

The overall risk of developing gestational trophoblastic neoplasia during the whole follow-up period after normalisation of hCG levels in <56 days of evacuation was substantially lower than in the group with normalisation of hCG levels in ≥56 days (posterior medians 0.06%, 95% credible interval 0.01% to 0.14% *v* 0.22%, 0.15% to 0.31%), with a posterior relative risk of 0.25 (0.06 to 0.72). Online supplemental figure 1 shows all posterior distributions.

**Figure 2:**
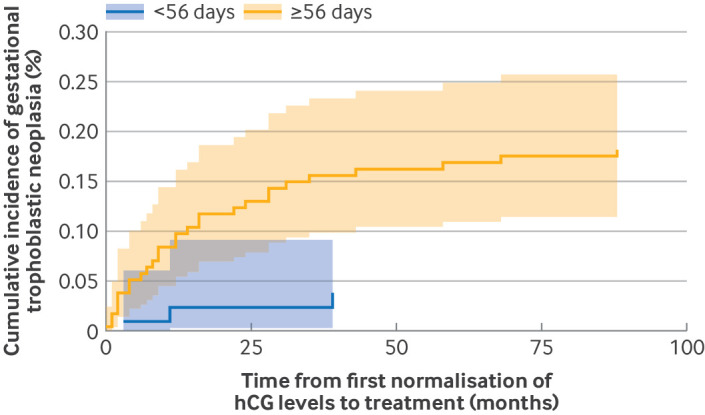
Cumulative incidence for development of gestational trophoblastic neoplasia over time after serum human chorionic gonadotropin (hCG) levels returned to normal (additional relapse risks are in figure 3). Time from first normalisation of hCG levels within the reference range of <56 or ≥56 days after uterine evacuation of a complete hydatidiform mole to treatment for gestational trophoblastic neoplasia. Solid lines represent the median of the posterior distribution for each patient group at each time interval. Shaded areas represent the corresponding 95% credible interval for each group at each time interval

The cumulative risk of developing gestational trophoblastic neoplasia in patients whose hCG levels normalised in <56 days increased minimally with time. Previous research^6^ focused on the clinically relevant time point of 56 days. Figures2  3 show the risk of developing gestational trophoblastic neoplasia over time for the two groups of patients: those who achieved normal serum levels of hCG in <56 or ≥56 days after uterine evacuation of a complete hydatidiform mole. If a patient had normal levels of hCG in <56 days, the risk of developing gestational trophoblastic neoplasia was small (0.04%, about 1 in 2619 patients) 39 months after normalisation of hCG levels. The equivalent risk for a patient who achieved normalisation of hCG levels ≥56 days was 0.16% (about 1 in 642 patients).

**Figure 3:**
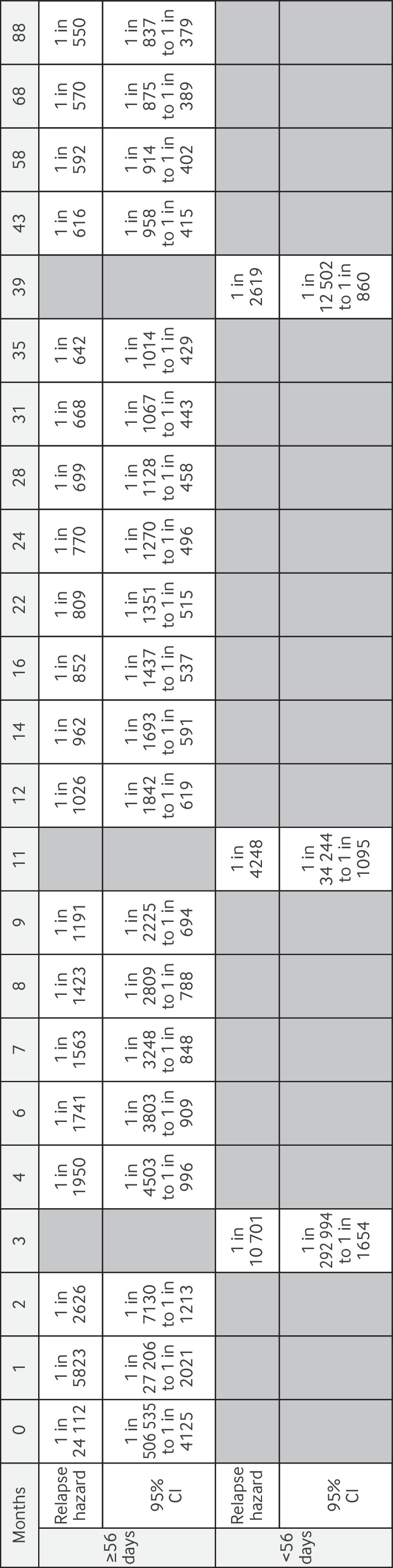
Risk of development of gestational trophoblastic neoplasia over time in patients whose serum human chorionic gonadotropin levels returned to normal in <56 or ≥56 days after uterine evacuation of a complete hydatidiform mole. Figure 2 shows cumulative incidence of developing GTN. CI, credible interval.

**Figure 4:**
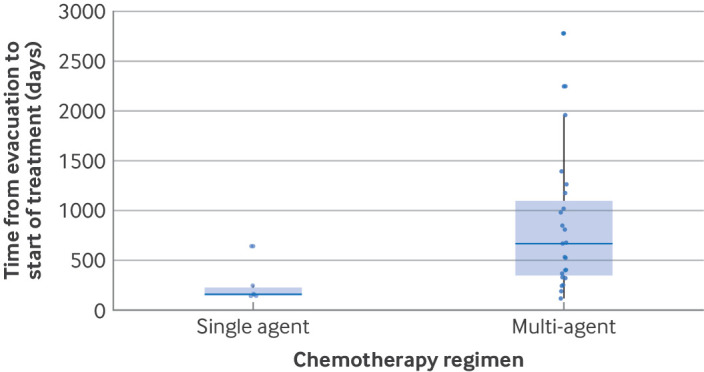
Time from uterine evacuation of a complete hydatidiform mole to treatment for gestational trophoblastic neoplasia by chemotherapy regimen in patients who developed gestational trophoblastic neoplasia after previous normalisation of serum human chorionic gonadotropin levels

Table 1 summarises the characteristics and clinical course of the 31 patients who developed gestational trophoblastic neoplasia after their serum hCG levels returned to normal. Most (n=20, 64.5%) presented with elevated hCG levels, and nine (29%) had vaginal bleeding. Eight patients (25.8%) had metastatic disease at presentation, including four (12.9%) with brain, five (16.1%) with lung, and two (6.5%) with vaginal metastases. Two patients (6.5%) were treated with surgery alone (one hysterectomy and one uterine evacuation) with no additional chemotherapy. For the remaining 29 patients, four received single agent chemotherapy and the remaining 25 required multidrug chemotherapy to achieve sustained remission. We hypothesised that the need for multi-agent chemotherapy might be greatest in patients whose tumours were detected after a longer period of time from the date of evacuation. Figure 4 shows that the median time from evacuation to treatment for gestational trophoblastic neoplasia was 367 days in patients who achieved remission after chemotherapy with a single agent compared with 524 days for patients requiring multi-agent chemotherapy for remission.

**Table 1:** Clinical and treatment characteristics of individual patients who developed gestational trophoblastic neoplasia after their serum levels of human chorionic gonadotropin had returned to normal

Patient No	Time from evacuation to first normal hCG level (days)	Gestational trophoblastic neoplasia						
		Time from first normal hCG level to treatment (days)	Presentation at time of diagnosis	FIGO score at diagnosis	Treatment	Time from end of treatment to last normal hCG level (years)	No of pregnancies (outcome) between initial complete hydatidiform mole and diagnosis	Genetics at time of diagnosis
**hCGnormalisationin** <**56 days**								
1	41	360	Pelvic pain, intraperitoneal bleeding from perforated uterus and haemorrhagic shock	5	Hysterectomy followed by EMACO×4	16.5	0	Matched original complete hydatidiform mole
2	47	114	Raised hCG, choriocarcinoma confirmed by pathology	4	MTX×7	23.6	0	Unknown
3	55	1209	Amenorrhoea, abdominal pain, lung and brain metastatic disease	11	High dose EMACO×6 and IT MTX×6	17.9	Unknown	Unknown
**hCGnormalisationin ≥56days**								
4	61	1334	Abdominal pain, raised hCG, uterine mass, and lung metastatic disease	10	EP induction then EMACO×8 with IT MTX×1	2.6	Unknown	Unknown
5	65	76	Raised hCG	2	MTX×6	13.0	0	Unknown
6	81	37	Raised hCG	2	MTX×1 switched to EMACO×7 because of steep increase in hCG	10.4	0	Unknown
7	83	2697	Vaginal bleeding with vaginal metastatic disease and choriocarcinoma	8	EMACO×9	27.9	1 pregnancy (term delivery)	Unknown
8	88	279	Unknown	4	EP×2, HuMMp×5	21.1	Unknown	Unknown
9	90	66	Vaginal bleeding, raised hCG	5	MTX×2, switched to EMACO×3 followed by hysterectomy	10.0	0	Unknown
10	96	438	Unknown	7	EMACO×10	36.0	Unknown	Unknown
11	105	875	Raised hCG after term pregnancy, imaging showed vascular lesion in uterus	7	MTX×4 switched to Dact×5 because of plateau and then EMACO×5	6.4	1 pregnancy (term delivery)	Unknown
12	105	1072	Raised hCG, opted for termination of pregnancy, no pregnancy tissue within uterus. Locally had 2 doses of methotrexate for pregnancy of unknown location. Persistently raised hCG	5* and 21†	MTX then IVA. Subsequent relapse 20 days after treatment completion. Declined subsequent treatment until stage IV disease then treated with induction EP, EMAEP, Pembro, and TETP	0.2	0	Unknown
13	108	739	Unknown	6	EP×2 and HuMMP×3. Subsequent relapse 44 days after treatment completion, treated with EMACO×8	0.06	Unknown	Unknown
14	109	138	Raised hCG	3	MTX×2, switched to VAC×3	30.4	0	Unknown
15	115	207	Raised hCG	6	MTX×2 switched to EP×4, switched to EMACO×2, switched to EMAEP×6 with hysterectomy in between	31.1	0	Matched original complete hydatidiform mole
16	115	290	Raised hCG, lung metastatic disease	3	EMAEP×2, followed by hysterectomy and thoracotomy, followed by TETP×2	17.8	1 pregnancy (non-molar abortion)	Invasive complete hydatidiform mole at relapse§
17	116	215	Unknown	5	EP and HuMMp×3	37.1	Unknown	Unknown
18	121	132	Vaginal bleeding, raised hCG, brain metastatic disease	10	EMACO×9 with IT MTX×3	20.3	0	Matched original complete hydatidiform mole
19	126	516	Raised hCG	4	MTX×3 switched to Dact×4 because of plateau	10.6	0	Unknown
20	127	267	Raised hCG, recurrent mass in uterus	7	Hysterectomy	0.6	0	Invasive complete hydatidiform mole at relapse§
21	131	60	Raised hCG	2	EMACO×6	5.0	0	Unknown
22	133	677	Vaginal bleeding, raised hCG	7	EMACO×9. Had a subsequent relapse 8 months after, treated with EMAEP and thoracotomy	0.3	Unknown	Unknown
23	140	878	Vaginal bleeding with neurological symptoms, brain, lung, and kidney metastatic disease	18	Induction EP, craniotomy for subarachnoid haemorrhage, high dose EMACO×7 and IT MTX×5	16.7	1 pregnancy (term delivery)	Unknown
24	141	4	Raised hCG	2	MTX×5	35.2	0	Unknown
25	151	373	Unknown	6	EP×2, switched to EMACO×2, CHAMOCA×2, EP×1	19.2	Unknown	Unknown
26	152	91	Raised hCG	2	EMACO×6	17.0	0	Unknown
27	174	493	Vaginal bleeding, lung and brain metastatic disease	9	EP induction then EMACO×6 with IT MTX×4	4.1	0	Unknown
28	174	2073	Vaginal bleeding with vaginal metastatic disease and choriocarcinoma	11	EP induction then EMACO×5 with IT MTX×3	5.0‡	Unknown	Unknown
29	194	1765	Vaginal bleeding, raised hCG, uterine evacuation, and placental site trophoblastic tumour	9	EMACO×7 switched to EMAEP×6	30.0	1 pregnancy (term delivery)	Unknown
30	222	953	Vaginal bleeding, raised hCG, uterine evacuation, and choriocarcinoma	5	Uterine evacuation, patient declined chemotherapy	0.3	Unknown	Matched original complete hydatidiform mole
31	306	371	Raised hCG	4	MTX×4, switched to EMACO×5. Subsequent relapse 41 days after treatment completion, treated with hysterectomy and EMAEP×2	0.1	0	Unknown

* Initial relapse.

† Start of Ttreatment initiation.

‡ Patient died of acute leukemialeukaemia.

§ Not compared with pregnancy at diagnosis.

CHAMOCA, cyclophosphamide, hydroxycarbamide, doxorubicin, dactinomycin, methotrexate, melphalan, and vincristine; Dact, dactinomycin; EMACO, etopiside, methotrexate, dactinomycin, cyclophosphamide, and vincristine; EMAEP, etopiside, methotrexate, dactinomycin, etopiside, and cisplatin; EP, etopiside and cisplatin; FIGO, International Federation of Gynaecology and Obstetrics; hCG, human chorionic gonadotropin; HuMMP, hydroxycarbamide, methotrexate, and mercaptopurine; IT, intrathecal; IVA, ifosfamide, vincristine, and dactinomycin; MTX, methotrexate; Pembro, pembrolizumab; TETP, paclitaxel, etopiside, paclitaxel, and cisplatin; VAC, vincristine, actinomycin, and cyclophosphamide.

We next examined if any pregnancies occurred between the diagnosis of complete hydatidiform mole and the development of gestational trophoblastic neoplasia. This information was important to exclude the possibility that the gestational trophoblastic neoplasia was a result of another pregnancy, different from the original complete hydatidiform mole. We found that 52% (16 of 31) of patients did not have any intervening pregnancies, but this information was unclear in 10 patients. Five patients had a pregnancy, four resulting in term deliveries and one in a non-molar pregnancy termination.

Genetic analysis was performed for six patients at the time of diagnosis of gestational trophoblastic neoplasia, one of whom had an interval pregnancy. In four of these patients (12.9%), we established that the subsequent gestational trophoblastic neoplasia was genetically derived from the original complete hydatidiform mole, but in two (6.5%) patients with an invasive complete hydatidiform mole, genetic analysis was not performed on the index mole because this material was no longer available. Therefore, in these two patients, a new gestational trophoblastic neoplasia unrelated to the original complete hydatidiform mole is possible, although one patient did not believe she had become pregnant again and the other had one interval non-molar termination.

## Discussion

### Principal findings

The overall risk of developing gestational trophoblastic neoplasia after normalisation of hCG levels was low (0.2%), with most patients receiving a diagnosis after the current six month hCG surveillance protocol. Moreover, we confirmed that the risk of gestational trophoblastic neoplasia was 3.6-fold lower in those whose hCG levels returned to normal in <56 days than in those with normalisation of hCG levels in ≥56 days (posterior medians 0.06%, 95% credible interval 0.01% to 0.14% *v* 0.22%, 0.15% to 0.31%), with a posterior relative risk of 0.25 (0.06 to 0.72). We have provided an overview of how the risk of gestational trophoblastic neoplasia evolves over time (figure 2), which could be used to estimate the risk in individual patients.

### Strengths and limitations of this study

This study’s strengths include that it was a large national population based cohort of women with a complete hydatidiform mole, so the risk of case ascertainment bias was low. During the study period, the guidelines for follow-up time were reduced from two years to six months. With the centralised care model, however, if patients presented again locally after their designated follow-up was complete, they were referred again to centralised care and therefore development of gestational trophoblastic neoplasia was recorded. In our analysis, we used the clinically relevant cut-off time of 56 days for normalisation of hCG levels because these patients underwent different lengths of hCG surveillance based on a previously identified risk for developing gestational trophoblastic neoplasia.^7 8^ Future work could consider time to normalisation of hCG levels as a continuous variable in the model to more rigorously determine the importance of time to normalisation and risk of developing gestational trophoblastic neoplasia.

Another limitation might have been follow-up time. The most recent patients had a diagnosis of complete hydatidiform mole pregnancy in November 2020, resulting in a follow-up period of two years. This short follow-up time could have underestimated the number of diagnoses of gestational trophoblastic neoplasia because 12 of 31 patients received a diagnosis 24 months after normalisation of hCG levels.

Also, patients who developed gestational trophoblastic neoplasia many months or years after a complete hydatidiform mole could have had an intervening causative pregnancy. This finding might have resulted in an overestimated risk of developing gestational trophoblastic neoplasia from the presumed causative complete hydatidiform mole. To mitigate this effect, genetic analysis proved that the gestational trophoblastic neoplasia was derived from the known complete hydatidiform mole in four of 29 patients. In the other two patients, where genetics showed that the gestational trophoblastic neoplasia material was a complete hydatidiform mole, we cannot be sure that this complete hydatidiform mole was derived from the original and not from a subsequent pregnancy. Nevertheless, this outcome seems unlikely in both patients, because one patient did not become pregnant again and the other had a verified non-molar pregnancy termination. Also, 16 of the remaining 25 patients with no genetic analysis denied any interval pregnancy. Consequently, in at least nine women, gestational trophoblastic neoplasia could have been derived from another pregnancy.

### Comparison with other studies

As previously reported, and similar to the findings in this study, the overall risk of developing GTN following hCG normalization is low^6^^15,18^. Monitoring of hCG levels after evacuation of a complete hydatidiform mole previously lasted for two years. Most current guidelines (American College of Obstetricians and Gynecologists, Royal College of Obstetricians and Gynaecologists, and International Federation of Gynaecology and Obstetrics), however, indicate that surveillance should continue for up to six months after normalisation of this hormone.^8^ In some countries,including the UK, if normalisation of hCG levels occurs in <56 days, follow-up is reduced to six months from the date of evacuation.^8^

### Policy Implications

Our findings support the idea that in patients whose hCG levels return to normal in <56 days, current UK and international practice could be changed to reduce monitoring protocols to one confirmatory normal hCG sample, to match current practice for partial hydatidiform moles.^9^ A recent Markov model based, cost effectiveness analysis in complete hydatidiform moles also suggested that it would be reasonable to reduce or potentially stop hCG monitoring after hCG levels have returned to normal.^19^

For women whose hCG levels return to normal in ≥56 days after a complete hydatidiform mole, how long should surveillance continue? Our study provides information for patients and clinicians to help determine what this interval should be. The risk data in figure 2 could be shared with patients for a personalised decision based process to decide when they feel it would be safe to stop hCG monitoring. In the UK, we have currently decided to continue offering up to six months of hCG monitoring after normalisation of hCG levels for these patients, but we might revise this approach after patient feedback. Clearly, many factors need to be considered in the decision process for affected women, including their reproductive plans and age, and whether a delayed diagnosis might result in a worse outcome. Although the absolute risk of recurrence remains low, patients need to be educated on the importance of seeking medical care even after completion of follow-up if they have abnormal bleeding or other concerning symptoms. A time interval of >2.8 years from the antecedent presumed causative pregnancy was found to correlate with worse outcomes in high risk gestational trophoblastic neoplasia.^20^ Fortunately, in our cohort, six of 31 patients had a diagnosis of gestational trophoblastic neoplasia >2.8 years after their initial complete hydatidiform mole, but this finding did not affect survival. We saw a trend for the need for much more aggressive combination chemotherapy, rather than chemotherapy with one drug, in patients who developed gestational trophoblastic neoplasia many months or years after their complete hydatidiform mole.

### Conclusions

In this study, we showed that the overall risk of developing gestational trophoblastic neoplasia in patients whose hCG levels had returned to normal in <56 days was low (posterior median 0.06%), which supports the idea that surveillance protocols can safely change to one confirmatory normal hCG value in this patient group. In those whose hCG levels return to normal in ≥56 days, figure 2 provides information for adequate counselling on the risk of developing gestational trophoblastic neoplasia over time and will inform shared decision making with patients and providers. Future research is needed to estimate the cost effectiveness and psychological effect of new follow-up recommendations.

## Supplementary material

10.1136/bmjmed-2024-001017 online supplemental file 1

10.1136/bmjmed-2024-001017 online supplemental file 2

10.1136/bmjmed-2024-001017 online supplemental file 3

## Data Availability

Data are available upon reasonable request.
