# Supplementary material for: Monitoring complete hydatidiform molar pregnancies after normalisation of human chorionic gonadotrophin: national retrospective population study
Source: BMJ Med. 2025 Apr 23;4(1):e001017. doi: 10.1136/bmjmed-2024-001017 (PMC12041671; doi:10.1136/bmjmed-2024-001017)
Supplement: online supplemental file 2 [file bmjmed-4-1-s002.pdf]

Supplementary figure 1

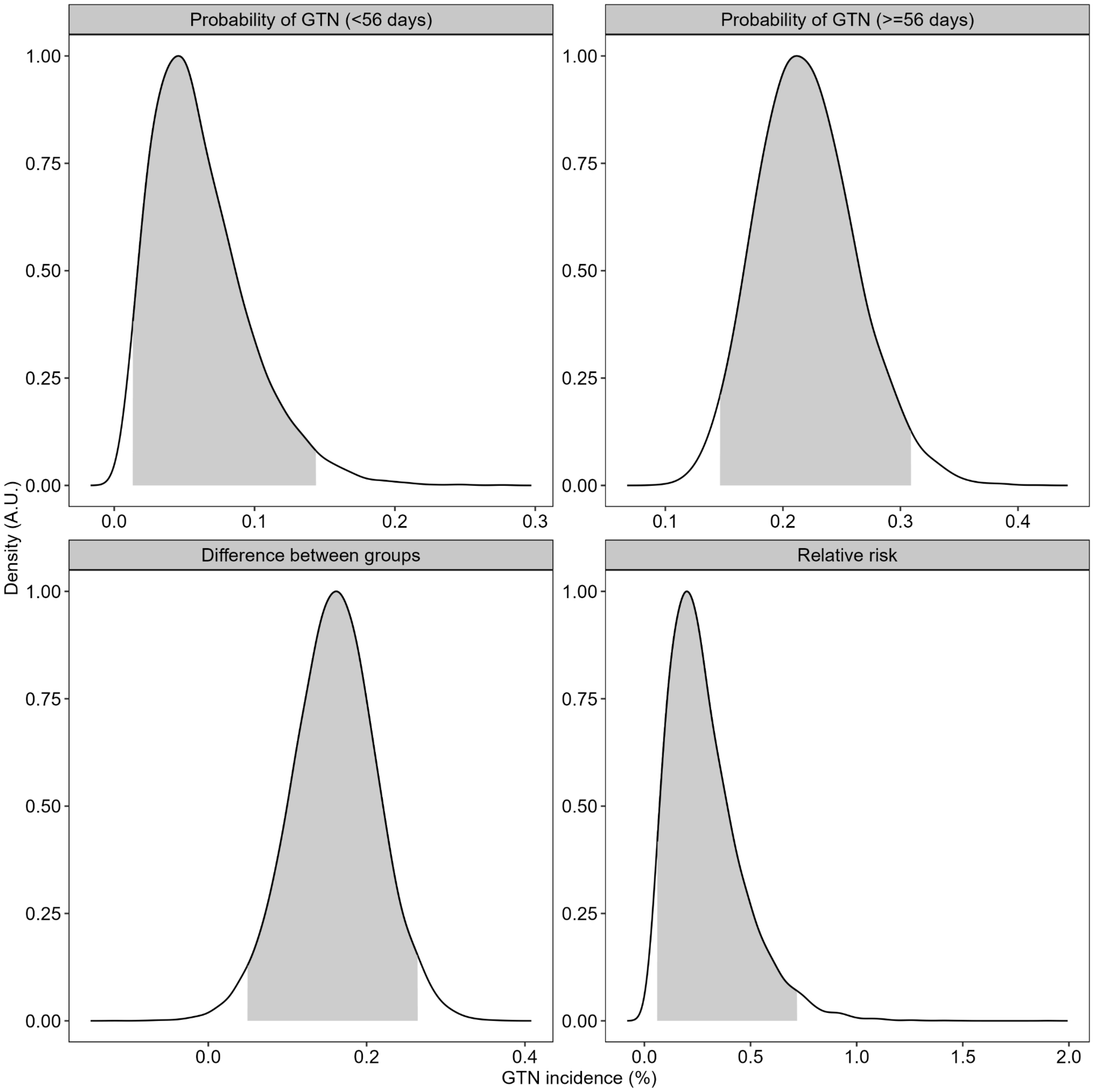

Fig S1. Posterior distributions for the model comparing the overall risk of developing GTN over the entire observation period. Black lines represent the density estimate of each posterior distribution across the 4000 Markov chain Monte Carlo iterations. Shaded areas represent the 95% credible intervals for each distribution.

Supplementary figure 2

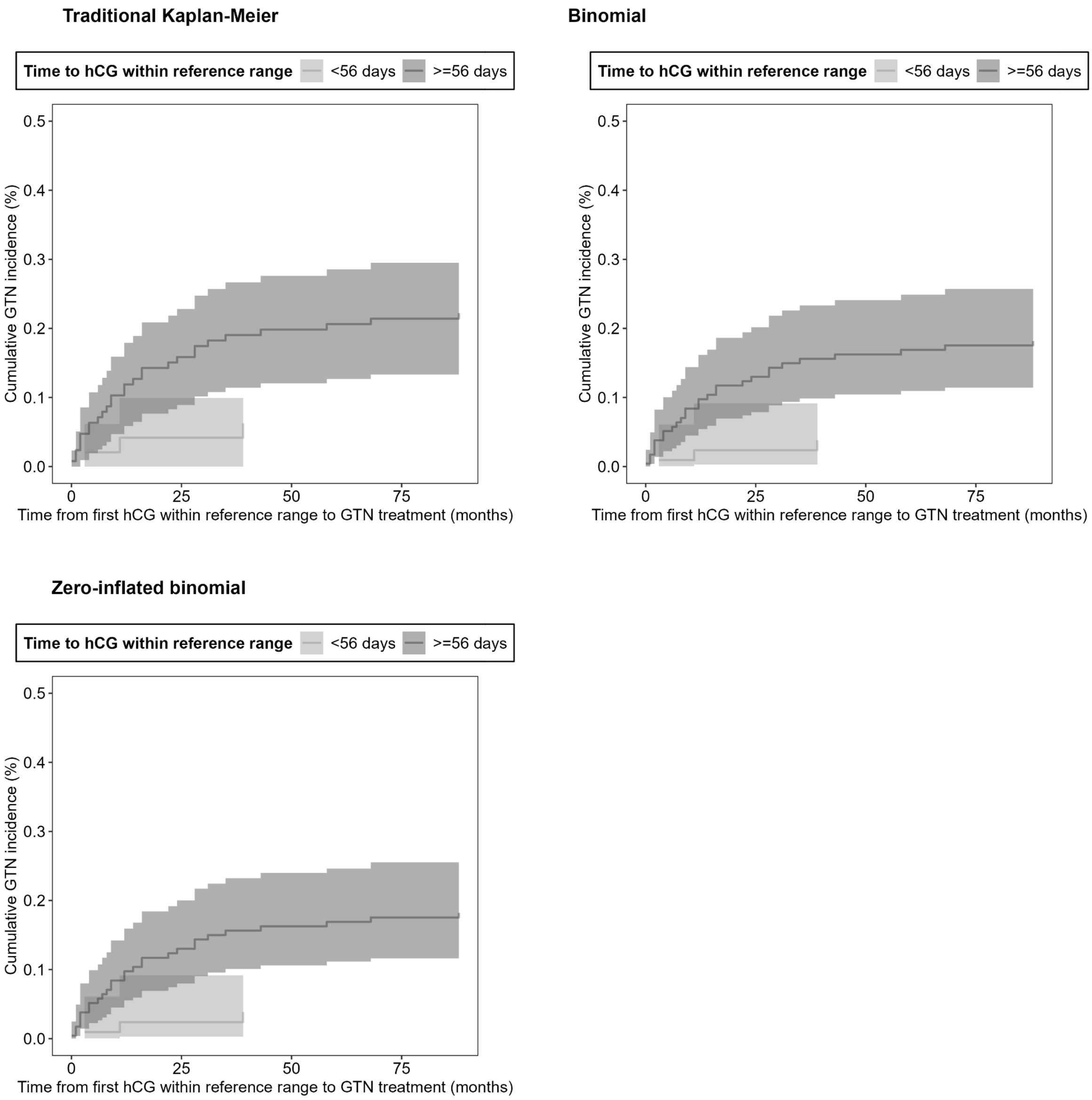

Figure S2. A comparison of different modelling approaches. Top-left, a traditional Kaplan-Meier model. Top-right, a Bayesian survival Bayesian model as specified in the text. Bottom-left a Bayesian zero-inflated binomial survival model fitted to the data. Shaded areas represent 95% confidence (Kaplan-Meier) or credible (Bayesian) intervals. Solid lines represent the point estimate (Kaplan-Meier) or median of the posterior (Bayesian) in each case.

Supplementary figure 3

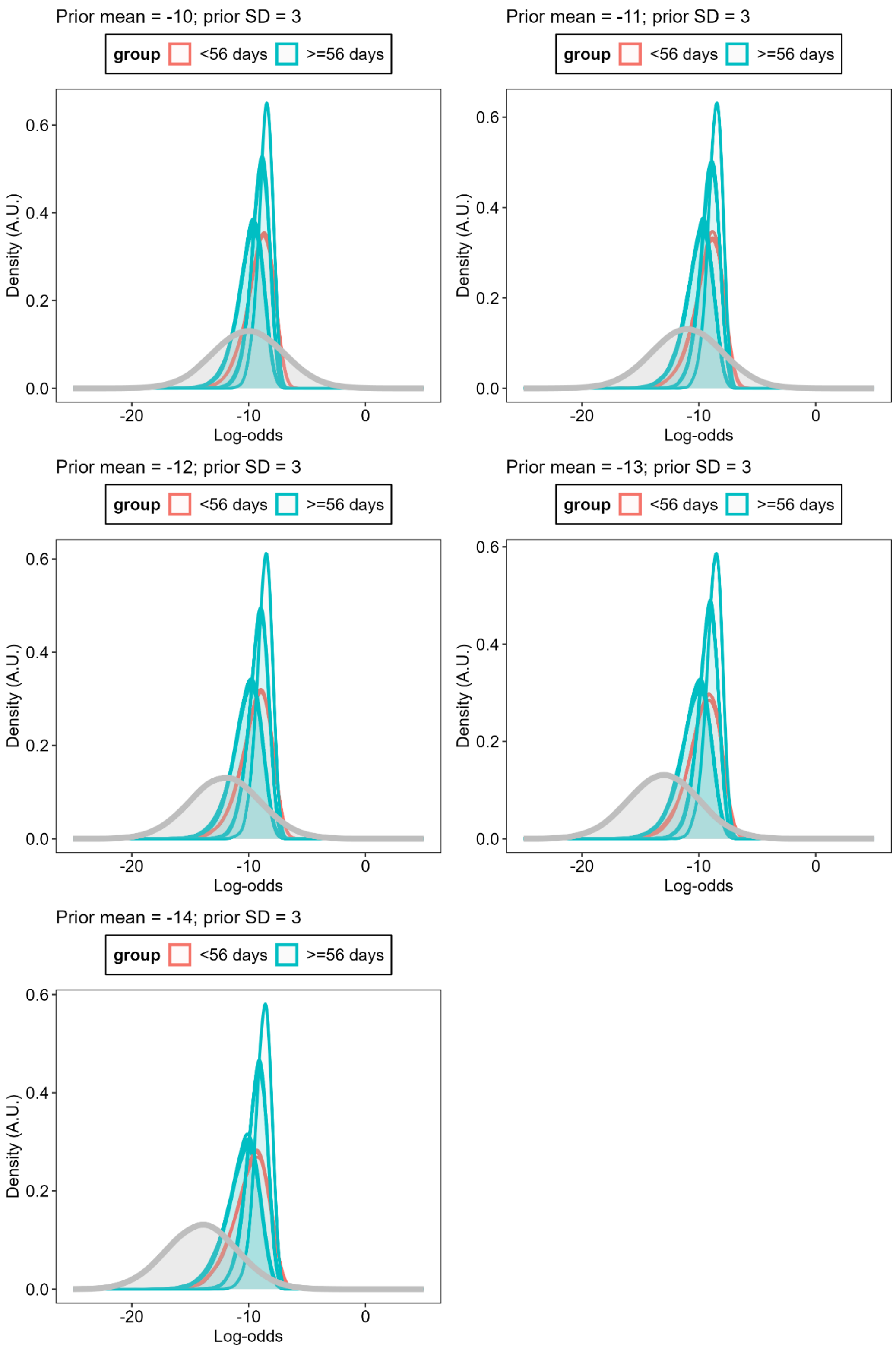

Figure S3. A comparison of priors for the Bayesian survival model. Prior distributions for the hazard at each time point are shown in grey and posterior distributions are coloured according to patient group, as indicated in the inset legends. Both types of distributions are shown in the log-odds scale as specified in the text.

Supplementary figure 4

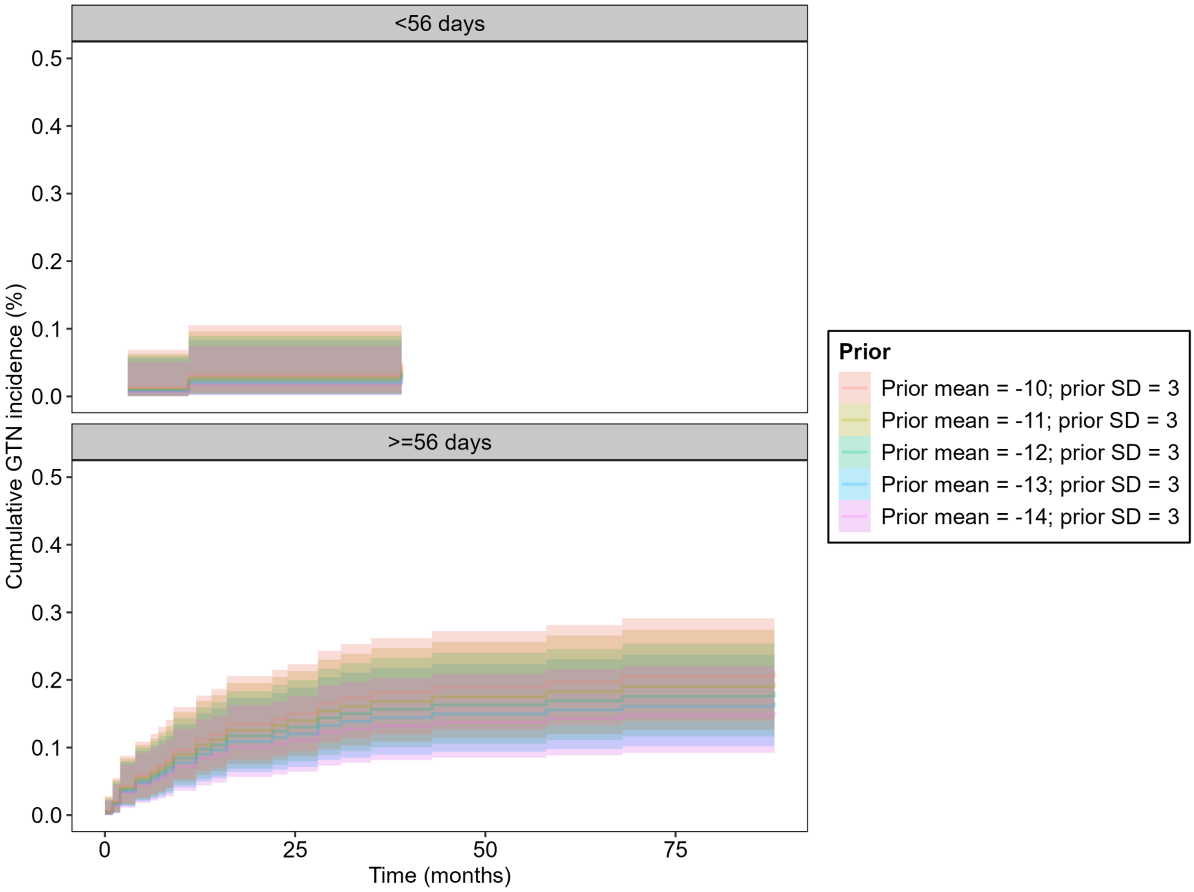

Figure S4. A comparison of priors for the Bayesian survival model – posterior distributions as cumulative incidence curves. Shaded areas represent 95% credible intervals. Solid lines represent the median of the posterior distribution in each case. The two patient groups are shown in separate boxes.
