# Supplementary material for: Monitoring complete hydatidiform molar pregnancies after normalisation of human chorionic gonadotrophin: national retrospective population study
Source: BMJ Med. 2025 Apr 23;4(1):e001017. doi: 10.1136/bmjmed-2024-001017 (PMC12041671; doi:10.1136/bmjmed-2024-001017)
Supplement: online supplemental file 3 [file bmjmed-4-1-s003.docx]

## Paper title: "Monitoring complete hydatidiform mole pregnancies after

## normalisation of human chorionic gonadotrophin levels: national

## retrospective population study"

## Journal: BMJ Medicine

## Code author: Ed Wilkes (edmund.wilkes@nhs.net)

## Date: 2024/10/30

## Packages ----

library(brms)

library(cowplot)

library(dplyr)

library(ggplot2)

library(ggsurvfit)

library(readxl)

library(survival)

library(survminer)

library(tidybayes)

library(tidyr)

plot_theme <- function(font_size) {

return(ggplot2::theme(

panel.background = element_blank()

,panel.grid.minor = element_blank()

,panel.grid.major = element_blank()

,axis.text = element_text(size = font_size, colour = "black")

,axis.title = element_text(size = font_size, colour = "black")

,panel.border = element_rect(fill = NA, colour = "black")

,strip.background = element_rect(fill = "grey80", colour = "black")

,strip.text = element_text(size = font_size, colour = "black")

,legend.background = element_rect(fill = NA, colour = "black")

,legend.title = element_text(face = "bold", size = font_size, colour = "black")

,legend.text = element_text(size = font_size, colour = "black"))

)

}

font_size <- 12

## GTD data (from Table 1) ----

df <- data.frame(

patient = 1:17424

,group = c(rep("<56 days", times = 4804), rep(">=56 days", times = 12620))

,event = c(

# <56 group

rep("GTN", times = 3)

,rep("Beyond monitoring", times = 4804 - 3)

# >=56 group

,c(rep("GTN", times = 28))

,rep("Beyond monitoring", times = 12620 - 28)

)

# 1 = event observed; 0 = no event observed within follow-up time (36 months)

,censor_variable = c(

# <56 group

rep(1, times = 3)

,rep(0, times = 4804 - 3)

# >=56 group

,rep(1, times = 28)

,rep(0, times = 12620 - 28)

)

,time_to_relapse = c(

# <56 group

c(114, 360, 1209)

,rep(1209, times = 4804 - 3)

# >=56 group

,c(4, 37, 60, 66, 76, 91, 132, 138, 207, 215, 267, 279, 290, 371, 373, 438, 493, 516, 677, 739, 875, 878, 953, 1072, 1334, 1765, 2073, 2697)

,rep(2697, times = 12620 - 28)

)

) |>

mutate(

months_to_relapse = as.integer(time_to_relapse / 30.417)

)

## Fit Bayesian model to the overall proportion/GTN incidence data ----

prior_mean <- -8

prior_sd <- 3

stanvars <- stanvar(prior_mean, "prior_mean")+

stanvar(prior_sd, "prior_sd")

model_all <- brm(

formula = censor_variable | trials(1) ~ 0 + group

,data = df

,family = binomial

,prior = c(

prior(normal(prior_mean, prior_sd), class = "b")

)

,chains = 4

,iter = 8000

,cores = 4

,seed = 1234

,refresh = 0

,stanvars = stanvars

)

df_posterior <- as_draws_df(model_all) |>

select(starts_with("b_")) |>

mutate(

iter = 1:16000

,`Probability of GTN (<56 days)` = plogis(`b_group<56days`) * 100

,`Probability of GTN (>=56 days)` = plogis(`b_group>EQ56days`) * 100

,`Difference between groups` = (`Probability of GTN (>=56 days)` - `Probability of GTN (<56 days)`)

,`Relative risk` = (`Probability of GTN (<56 days)` / `Probability of GTN (>=56 days)`)

) |>

pivot_longer(-iter) |>

group_by(name) |>

mutate(median = median(value), lwr = quantile(value, 0.025), upr = quantile(value, 0.975)) |>

reframe(

density_x = density(value, adjust = 1.5)$x

,density_y = density(value, adjust = 1.5)$y

,median = unique(median)

,lwr = unique(lwr)

,upr = unique(upr)

,max_density = max(density_y, na.rm = TRUE)

,density_y_norm = density_y / max_density

) |>

rowwise() |>

mutate(density_x_95 = if_else(density_x > lwr & density_x < upr, true = density_x, false = NA)) |>

filter(!grepl("^b_*", name)) |>

mutate(

name = factor(

name

,levels = c(

"Probability of GTN (<56 days)"

, "Probability of GTN (>=56 days)"

,"Difference between groups"

,"Relative risk"

)

)

)

p_model_all <- ggplot(

df_posterior |> filter(!grepl("^b_*", name))

,aes(x = density_x, y = density_y_norm)

)+

geom_line()+

geom_area(aes(x = density_x_95), fill = "grey", alpha = 0.75, colour = "black")+

facet_wrap(~name, scales = "free", nrow = 2)+

plot_theme(font_size = 12)+

xlab("GTN incidence (%)")+

ylab("Density (A.U.)")

p_model_all

ggsave("FigS1_model_all_posteriors.png", dpi = 300, width = 10, height = 10)

# Get posterior summaries

df_posterior |>

group_by(name) |>

summarise(median = unique(median), lwr = unique(lwr), upr = unique(upr))

# name median lwr upr

# Probability of GTN (<56 days) 0.055 0.013 0.144

# Probability of GTN (>=56 days) 0.218 0.147 0.309

# Difference between groups 0.160 0.050 0.265

# Relative risk 0.252 0.059 0.722

## Fit Bayesian survival model (Figure 2) ----

# Gather survival summary

km_summary <- survival::survfit(

Surv(months_to_relapse, censor_variable) ~ group

,data = df

,conf.int = 0.95

) |> summary()

df_model <- tibble(

months = km_summary$time

,n_risk = km_summary$n.risk

,n_events = km_summary$n.event

,cum_haz = km_summary$cumhaz

,lower = -log(km_summary$upper)

,upper = -log(km_summary$lower)

,group = c(rep("<56 days", 3), rep(">=56 days", 20))

) |>

group_by(group) |>

mutate(

`p(t)` = n_events / n_risk

,n_censored = n_risk - n_events - lead(n_risk, default = 0)

,interval = 1:n()

,interval_f = factor(1:n(), levels = 0:n())

,start = months

,end = lead(months, default = Inf)

) |>

select(interval:interval_f, months, start:end, n_risk:n_events, n_censored, `p(t)`)

# Fit the time-to-event Kaplan-Meier model

prior_mean <- -12

prior_sd <- 3

stanvars <- stanvar(prior_mean, "prior_mean")+

stanvar(prior_sd, "prior_sd")

model <- brms::brm(

n_events | trials(n_risk) ~ 0 + interval_f:group

,data = df_model

,family = binomial()

,prior = c(

prior(normal(prior_mean, prior_sd), class = "b")

)

,chains = 4

,iter = 8000

,cores = 4

,seed = 1234

,control = list(adapt_delta = 0.8)

,stanvars = stanvars

)

# Get model outputs

getModelOutputs <- function(model) {

df_draws <-

as_draws_df(model) |>

select(starts_with("b_")) |>

mutate_all(inv_logit_scaled) |>

mutate(iter = 1:n()) |>

pivot_longer(

-iter

,names_to = "interval:group"

,values_to = "p"

) |>

mutate(

group = rep(c("<56 days", ">=56 days"), each = 20, times = 16000)

,interval = rep(1:20, times = 32000)

) |>

arrange(interval) |>

group_by(iter, group) |>

mutate(survival = cumprod(1 - p), hazard = -log(survival)) |>

ungroup()

# Summarise and plot the outputs

df_plot <- left_join(df_model, df_draws, by = c("interval", "group"))

df_bayes_summary <- df_plot |>

group_by(group, interval, months) |>

summarise(

naive_prob_estimate = unique(`p(t)`)

,median_prob = median(p)

,lwr_prob = quantile(p, 0.025)

,upr_prob = quantile(p, 0.975)

,survival = median(survival)

,median_hazard = median(hazard)

,lwr_hazard = quantile(hazard, 0.025)

,upr_hazard = quantile(hazard, 0.975)

,hazard_nat = round(1 / median_hazard, 0)

,lwr_hazard_nat = round(1 / lwr_hazard, 0)

,upr_hazard_nat = round(1 / upr_hazard, 0)

) |>

mutate(

median_hazard = round(median_hazard * 100, 2)

,lwr_hazard = round(lwr_hazard * 100, 2)

,upr_hazard = round(upr_hazard * 100, 2)

)

p <- ggplot(df_plot, aes(x = months, y = hazard * 100, colour = group, fill = group))+

stat_lineribbon(step = "hv", linewidth = 3/4, .width = 0.95, alpha = 0.35, point_interval = "median_qi")+

theme_bw()+

labs(

x = "Time from first hCG within reference range to GTN treatment (months)"

,y = "Cumulative GTN incidence (%)"

)+

theme(legend.position = "top")+

ylim(c(0, 0.5))+

plot_theme(font_size)+

labs(fill = "Time to hCG within reference range", colour = "Time to hCG within reference range")+

scale_fill_manual(values = c("grey50","grey10"))+

scale_colour_manual(values = c("grey50", "grey10"))

return(list(data = df_plot, summary = df_bayes_summary, plot = p))

}

model_output <- getModelOutputs(model)

# Figure 2

model_output$plot

ggsave("Fig2_survival_curves.png", dpi = 300, width = 16, height = 8)

## Days from evacuation to treatment (Figure 3) ----

df_chemo <- read_excel("~/Boxplot_data_20241023.xlsx") |>

filter(`single agent=1\nmulti-agent=2\nsurgery=0` %in% c(1,2)) |>

mutate(

chemo = factor(

case_when(

`single agent=1\nmulti-agent=2\nsurgery=0` == 1 ~ "Single agent"

,`single agent=1\nmulti-agent=2\nsurgery=0` == 2 ~ "Multi-agent"

)

,levels = c("Single agent", "Multi-agent")

)

)

p_boxplot <- ggplot(

df_chemo

,aes(

x = chemo

,y = `Days from evacuation to treatment`

,fill = chemo

)

)+

geom_boxplot(alpha = 0.5)+

geom_jitter(width = 0.025, alpha = 0.5)+

scale_fill_manual(values = c("grey50","grey10"))+

plot_theme(font_size = 12)+

xlab("Chemotherapy strategy given")+

ylab("Days from evacuation to treatment initiation")+

theme(legend.position = "none")+

scale_y_continuous(breaks = seq(from = 0, to = 3000, by = 500))

p_boxplot

ggsave("Fig3_boxplot.png", dpi = 600, width = 10, height = 8)

## Reviewer response: compare Kaplan-Meier vs Bayesian models ----

model_zib <- brms::brm(

n_events | trials(n_risk) ~ 0 + interval_f:group

,data = df_model

,family = zero_inflated_binomial()

,prior = c(

prior(normal(prior_mean, prior_sd), class = "b")

)

,chains = 4

,iter = 8000

,seed = 1234

,cores = 4

,control = list(adapt_delta = 0.9, max_treedepth = 15)

,stanvars = stanvars

)

loo_compare(add_criterion(model, "loo"), add_criterion(model_zib, "loo"))

# elpd_diff se_diff

# add_criterion(model, "loo") 0.0 0.0

# add_criterion(model_zib, "loo") -1.8 0.8

model_output_zib <- getModelOutputs(model_zib)

p_km <- tibble(

months = km_summary$time

,n_risk = km_summary$n.risk

,n_events = km_summary$n.event

,cum_haz = km_summary$cumhaz

,lower = -log(km_summary$upper)

,upper = -log(km_summary$lower)

,group = c(rep("<56 days", 3), rep(">=56 days", 20))

) |>

ggplot(aes(x = months, y = cum_haz * 100, colour = group, fill = group))+

geom_lineribbon(step = "hv", linewidth = 3/4, aes(ymin = lower * 100, ymax = upper * 100), alpha = 0.35)+

theme_bw()+

labs(

x = "Time from first hCG within reference range to GTN treatment (months)"

,y = "Cumulative GTN incidence (%)"

)+

theme(legend.position = "top")+

ylim(c(0, 0.5))+

plot_theme(font_size)+

labs(fill = "Time to hCG within reference range", colour = "Time to hCG within reference range")+

scale_fill_manual(values = c("grey50","grey10"))+

scale_colour_manual(values = c("grey50", "grey10"))

plot_grid(

p_km, model_output$plot, model_output_zib$plot

,labels = c("Traditional Kaplan-Meier", "Binomial", "Zero-inflated binomial")

,scale = 0.85

)

ggsave("FigS2_comparing_models.png", dpi = 300, width = 12, height = 12)

## Reviewer response: prior sensitivity analysis ----

# Different prior distributions' parameters

df_priors <- data.frame(

prior = 1:5

,mean = -10:-14

,sd = 3

)

list_priors <- split(df_priors, f = df_priors$prior)

# Fit models

list_models <- lapply(

list_priors

,function(prior) {

stanvars <- stanvars <- stanvar(prior$mean, "prior_mean")+

stanvar(prior$sd, "prior_sd")

model <- brms::brm(

n_events | trials(n_risk) ~ 0 + interval_f:group

,data = df_model

,family = binomial()

,prior = c(

prior(normal(prior_mean, prior_sd), class = "b")

)

,chains = 4

,iter = 4000

,cores = 4

,seed = 1234

,control = list(adapt_delta = 0.8)

,stanvars = stanvars

)

return(model)

}

)

# Plot priors vs posteriors for each model

list_prior_plots <- lapply(

seq_along(list_models)

,function(x) {

df_priors <- data.frame(

prior_log_odds = rnorm(

n = 100000

,mean = list_priors[[x]]$mean

,sd = list_priors[[x]]$sd)

)

df_draws <-

as_draws_df(list_models[[x]]) |>

select(starts_with("b_")) |>

mutate(iter = 1:n()) |>

pivot_longer(

-iter

,names_to = "interval:group"

,values_to = "posterior_log_odds"

) |>

mutate(

group = rep(c("<56 days", ">=56 days"), each = 20, times = 8000)

,interval = rep(1:20, times = 16000)

) |>

filter((group != "<56 days" | interval <= 3))

p <- ggplot()+

geom_density(

data = df_draws

,aes(x = posterior_log_odds, group = `interval:group`, colour = group, fill = group)

,alpha = 0.02

,adjust = 2

,linewidth = 1

)+

geom_density(

data = df_priors

,aes(x = prior_log_odds)

,colour = "grey"

,fill = "grey"

,alpha = 0.25

,adjust = 2

,linewidth = 2

)+

xlim(c(-25, 5))+

plot_theme(font_size = 12)+

xlab("Log-odds")+

ylab("Density (A.U.)")+

theme(legend.position = "top")+

ggtitle(paste0("Prior mean = ", list_priors[[x]]$mean, "; prior SD = ", list_priors[[x]]$sd))

return(p)

}

)

plot_grid(plotlist = list_prior_plots, ncol = 2)

width <- 9

ggsave("FigS3_Posterior_vs_priors.png", dpi = 300, width = width, height = width * 1.5)

# Plot the hazard curves overlaid

df_plots_all <- lapply(

seq_along(list_models)

,function(x) {

df_draws <-

as_draws_df(list_models[[x]]) |>

select(starts_with("b_")) |>

mutate_all(inv_logit_scaled) |>

mutate(iter = 1:n()) |>

pivot_longer(

-iter

,names_to = "interval:group"

,values_to = "p"

) |>

mutate(

group = rep(c("<56 days", ">=56 days"), each = 20, times = 8000)

,interval = rep(1:20, times = 16000)

) |>

arrange(interval) |>

group_by(iter, group) |>

mutate(

survivor = cumprod(1 - p)

,hazard = -log(survivor)

,prior = paste0("Prior mean = ", list_priors[[x]]$mean, "; prior SD = ", list_priors[[x]]$sd)

) |>

ungroup()

df_plot <- left_join(df_model, df_draws, by = c("interval", "group"))

return(df_plot)

}

) |> bind_rows()

p_all_scaled <- ggplot(df_plots_all, aes(x = months, y = hazard * 100, colour = prior, fill = prior))+

stat_lineribbon(step = "hv", size = 3/4, .width = 0.95, alpha = 0.25, point_interval = "median_qi")+

plot_theme(font_size = 12)+

labs(

x = "Time (months)"

,y = "Cumulative GTN incidence (%)"

)+

theme(legend.position = "right")+

facet_wrap(~group, ncol = 1)+

ylim(c(0, 0.5))+

labs(colour = "Prior", fill = "Prior")

p_all_scaled

ggsave("FigS4_Hazard_curves_overlaid.png", dpi = 300, width = width, height = width * 0.75)
