# Supplementary material for: Monitoring complete hydatidiform molar pregnancies after normalisation of human chorionic gonadotrophin: national retrospective population study
Source: BMJ Med. 2025 Apr 23;4(1):e001017. doi: 10.1136/bmjmed-2024-001017 (PMC12041671; doi:10.1136/bmjmed-2024-001017)
Supplement: Supplementary file 4 [file bmjmed-2024-001017.reviewer_comments.pdf]

PEER REVIEW HISTORY

BMJ Medicine publishes all reviews undertaken for accepted manuscripts. Reviewers are asked to complete a checklist review form and are provided with free text boxes to elaborate on their assessment. These free text comments are reproduced below.

ARTICLE DETAILS

**TITLE (PROVISIONAL)**	Is it time to stop monitoring a complete hydatidiform mole pregnancy after hCG normalization? A national retrospective population study
**AUTHORS**	Coopmans, Leonora; Swift, Brenna; Singh, Kam; Coyle, Christopher; Wilkes, Edmund; Jabbar, Imran; Maher, Geoffrey; Aguiar, Xianne; Salman, Lina; Palmer, Julia; Sarwar, Naveed; Caldwell, Reece; Ghorani, Eshan; Kaur, Baljeet; van Trommel, Nienke; Lok, Christianne; Winter, Matthew; Seckl, Michael

VERSION 1 - REVIEW

**REVIEWER NAME**	Shorter, Jade
**REVIEWER AFFILIATION**	Stanford University, OBGYN
**REVIEWER CONFLICT OF INTEREST**	None
**DATE REVIEW RETURNED**	02-Aug-2024

**GENERAL COMMENTS**	Thank you for the opportunity to review the manuscript: “Is it time to stop monitoring a complete hydatidiform mole pregnancy after hCG normalization? A national retrospective population study.” This study addresses an important issue regarding appropriate follow-up for patients with a complete hydatidiform mole pregnancy. This study helps to fill in a gap in the literature regarding the risk of transformation to GTN if hCG values normalize within 56 days. Evidence that supports decreasing the burden of follow-up for these patients will help improve patient satisfaction and support a patient’s desire for attempting to conceive soon after this challenging diagnosis.<br><br>My specific comments are noted below:<br><br>Introduction:<br><br>1. Introduction is well written and helps to frame the importance of the topic and gaps in current literature.<br><br>Methods:<br><br>1. A major strength of this study is the large national population over a 40 year time period<br><br>2. Page 11, paragraph 2, lines 25-29: Time to diagnosis of GTN was from the date of normal hCG to the start of treatment. Why was the definition not from the date of normal hCG until date of diagnosis? Was the definition chosen used in prior studies?<br><br>Results:<br><br>1. Table and results are clearly presented.

	2. Authors provided detailed information on patients who developed GTN, however some information limited by retrospective nature of study<br><br>3. Interval pregnancy history noted with pregnancy outcomes for those patients who developed GTN. It would be interesting to know the overall pregnancy outcomes for patients without development of GTN. Specifically, did any patients become pregnant prior to completing the recommended time for hCG surveillance and what were the outcomes?<br><br>4. Table 1: Many patients' presentation at the time of GTN diagnosis was only an elevated hCG. Do you have data on why the hCG level was tested? Was it due to: missed menses? symptoms of pregnancy? Incidental testing? Often we counsel patients about symptoms of abnormal vaginal bleeding, but knowing the context of why an hCG was collected could help with counseling patients for precautions for GTN.<br><br>Discussion:<br><br>1. The data is compelling for decreasing monitoring of hCG surveillance after a complete hydatidiform mole pregnancy<br><br>2. Agree that this study can help guide shared decision making for the length of monitoring of hCG if normalization > 56 days.

**REVIEWER NAME**	Albright, Benjamin
**REVIEWER AFFILIATION**	UNC, Obstetrics and Gynecology
**REVIEWER CONFLICT OF INTEREST**	I am a lead author of some related studies on this topic.
**DATE REVIEW RETURNED**	23-Aug-2024

**GENERAL COMMENTS**	Major comments:<br>- This study is a retrospective cohort study using the UK databases for molar pregnancy to evaluate the specific question of the role of ongoing hCG screening after normalization for patients with complete mole<br>- The data utilized is an excellent resource for this question. The paper is generally clear, concise, and well written. The methodology appears appropriate to my level of statistical training, though I am unfamiliar with the Bayesian statistical modeling and defer to a statistical reviewer to comment on this choice<br>- Generally, I do think this data likely warrants publication, but the authors need to use caution in how the frame the novelty of the data and of the conclusion. The authors state, "In keeping with prior data7,8, patients with CHM who achieved hCG normalization within 56 days had a significantly lower risk of GTN compared to those normalizing beyond this (1/1159 vs 1/308, OR 0.27 95% CI 0.08–0.88, p=0.03). Consequently, patients with CHM appear to need longer hCG monitoring if the hCG normalizes beyond 56 days from the date of uterine evacuation." This data is drawn from Coyle et al (reference 6), then just a few sentences later state "However, no data are currently available to inform us of the relative risk of malignant transformation of CHM when the hCG normalizes either within or beyond 56 days." Coyle et al includes Charing Cross cases of CHM and PHM from 1980 to 2009, whereas the current study

includes Charing Cross and Sheffield cases from 1980-2020. Therefore, my interpretation is that the current study includes the 8,400 CHM from the Coyle et al study, plus about 9000 additional cases from Sheffield or more recent years at Charing Cross. It is inaccurate to state that there are no data about relative risk with normalization within or beyond 56 days. This is reported in Coyle et al, and this topic is also assessed in the systematic review by Albright, et al, which compiles Coyle et al with data from other trophoblastic disease centers in the US (NETDC) and other countries. The conclusion of this paper is not truly novel as it is highly similar to the conclusions from both Coyle et al and Albright et al.

- I think the detailed description of cases with GTN after hCG normalization is actually the most novel part of the study, though it is framed as a secondary aim of the analysis. I would recommend considering emphasizing this part of the analysis more in the objectives and abstract.
- Related to the above point regarding data overlap, I am trying to correlate the Coyle et al data with the data in the current paper. Coyle et al reports 3 GTN cases among 3,477 CHM with normalization <56 days, and 23 GTN cases among 7,081 CHM with normalization >56 days. This paper reports 3/4804 with normalization <56 days, and 28/12,620 cases with normalization >56 days. This implies that the addition of 11 years of follow up from Charing Cross and 30 years of data from Sheffield added no cases of GTN among ~1400 added cases with normalization <56 days, and 5 cases of GTN among ~5000 added cases with normalization >56 days. The low number of cases from the added data is somewhat surprising, but it may be accurate. I just ask that the authors re-confirm the accuracy of this data and this comparison to the Coyle et al study. I think it would be useful and informative as a sensitivity analysis to describe the data from the two centers separately for reference, as any data from Sheffield is novel and not included in the previously published Coyle et al paper. I don't have any reason to suspect differences in these populations, but it seems like the rates may have been different between the two centers potentially?
- "Figure 3 shows a non-significant trend in this direction with the median time being 367 days in the single-agent group and 524 days in the multi-agent group (p=0.811)" -> a p value of 0.811 is not much of a trend, would use caution with this framing. It would be more accurate to state that there was no statistically significant difference and state medians and p value, allow the reader to interpret beyond that.
- I think it would be beneficial for the discussion section to reference and help frame the data with the findings from https://www.ncbi.nlm.nih.gov/pmc/articles/PMC9941751/ (of which I am an author) which is a cost-effectiveness analysis assessing this exact question of the optimal surveillance strategy. The data is applicable to this topic/discussion.
- I agree with the author's conclusions that the surveillance guidelines for complete mole could likely be further adjusted based on this and related data. I do think it would be helpful to frame this recommendation by describing in more detail how recommendations for surveillance have already evolved in recent years from the traditional 6 months or more of follow up for partial and complete mole to the current guidelines by RCOG and FIGO/ACOG.

Minor comments:

- Many of the references in the reference list are duplicated and

	given 2 different numbers (6=26, 7-27, 18=36 for example).<br>- In the introduction, when describing the typical course of molar pregnancy, I think it is worth mentioning that the natural course of a molar pregnancy is likely to result in spontaneous abortion and some molar pregnancies may be unrecognized early spontaneous abortions.<br>- "In contrast, for CHM we noted a slightly higher risk of post-molar GTN after hCG normalization (0.24%, n=20/8400) which decreased from 1/839 at 4 months to 1/1677 patients at 12 months after the 1st normal hCG value6. -> would be helpful to put the percentages rather than fractions for easier comparisons between the values at the different time points. This same point about putting percentages in addition to fractions applies to the results section as well.

**REVIEWER NAME**	Riley, Richard
**REVIEWER AFFILIATION**	University of Birmingham, Institute of Applied Health Research
**REVIEWER CONFLICT OF INTEREST**	None
**DATE REVIEW RETURNED**	29-Sep-2024

**GENERAL COMMENTS**	Thank you for the opportunity to review this interesting paper. It is clear the authors have worked hard to produce this research, examining a rare event from 40 years of data. I have some comments from a statistical point of view.<br><br>1) Abstract says: 'The risk of developing GTN following uterine evacuation' – here and in the whole article, I am looking for the time-point of interest. Risk by when, exactly?<br>2) Mantel Haenzel tests and relative risks are derived, but these approaches assume (I think) the data are binary outcomes without censoring before the time-point of interest for the event risk to be defined by. But surely there is censoring due to loss to follow-up (i.e. different lengths of follow-up need to be accounted for) over time, such that not all individuals event status is known by the time point of interest?<br>3) A related point is that equations 1 to 3 shown a 'time to event' model but this is not actually accounting for censoring (which the name 'time to event' model might usually imply) but simply is a logistic regression model with an interaction with time.<br>4) I am confused as to why time is categorised. Surely a more complete picture is to model the log-odds of event as a function of time, allowing for non-linear relationship with say splines?<br>5) I have no issue with taking a Bayesian approach, or the use of informative prior distributions, but here I did wonder whether the prior distribution is dominating the data given how sparse the outcome is. The prior given seems to be specified on the log odds of the event being low, but I cannot see what the prior is on the actual effect (Relative risk) of interest, or how informative this might be. Indeed, in equation (2), it looks like this is avoided by modelling the log-odds for each group*time separately, and with the prior the same on the log-odds for each; thus am I right that this is equivalent to assuming no effect (difference) between groups a priori? Post-estimation, I assume a function of the alpha_ij terms are then used to make inferences about differences in effect?<br>6) The results are given as risks and relative risks, and yet the actual Bayesian model is specified in terms of odds<br>7) Is it time after the test result? Or the time after the uterine evacuation that is being modelled?

	8) If GTN occurred before the test result – but then later there was a normalization test result – how was this handled in the analysis? The patients were excluded I think, so the results only apply to those for whom GTN had not occurred before hand. This needs to be made clearer. A broader question is whether we have a somewhat selective group of individuals who can end up with a normal test result (perhaps that does not even matter). For example, one person died of a different (?) issue – who were they handled in the analysis?<br>9) P-values are provided and yet a Bayesian approach is taken, which are not compatible.<br>10) Given that the study spans 40 years, I did expect to see some examination of the impact of years from 1980 to 2020. Could there be confounding by year? For example, if testing is delayed since 2000 (so more normal tests post 56 days), and the incidence of the outcome is gradually growing over time too.<br>11) Relative risks and CIs should always be given for each comparison (in addition to the risks by each group separately)<br>12) 'Figure 3 shows a non significant trend in this direction with the median time being 367 days in the single-agent group and 524 days in the multi-agent group (p=0.811).' – this seems a strong emphasis without any statistical justification. Just say further research is needed on this.<br>13) Another broad question is why the emphasis on 56 days? Why not 28 days or 74 days? It would have been helpful to examine this without categorisation, to see if 56 days is truly the best place for this subjective cut-off.<br><br>In summary, there are a number of areas for revision and clarification, before BMJ Medicine can evaluate this further. Some of queries may reflect a lack of clinical understanding of this particular area, but generally I hope my comments are constructive for the authors moving forwards.

VERSION 1 – AUTHOR RESPONSE

We would like to thank the reviewers for their comments

Reviewer: 1

Comments to the Author

Thank you for the opportunity to review the manuscript: "Is it time to stop monitoring a complete hydatidiform mole pregnancy after hCG normalization? A national retrospective population study." This study addresses an important issue regarding appropriate follow-up for patients with a complete hydatidiform mole pregnancy. This study helps to fill in a gap in the literature regarding the risk of transformation to GTN if hCG values normalize within 56 days. Evidence that supports decreasing the burden of follow-up for these patients will help improve patient satisfaction and support a patient's desire for attempting to conceive soon after this challenging diagnosis.

My specific comments are noted below:

Introduction:

1. Introduction is well written and helps to frame the importance of the topic and gaps in current literature.

Methods:

1. A major strength of this study is the large national population over a 40 year time period

2. Page 11, paragraph 2, lines 25-29: Time to diagnosis of GTN was from the date of normal hCG to the start of treatment. Why was the definition not from the date of normal hCG until date of diagnosis? Was the definition chosen used in prior studies?

- In the UK, patients diagnosed with GTN start their treatment on the same or the next day at the latest. Our database records the treatment start date so this is why we used this in the manuscript. We have modified the sentence in the methods section to reflect this.

  'The time to diagnosis of GTN was determined from the date of the first normal hCG to the start of treatment for GTN which in the UK is the same day as the diagnosis.'

Results:

1. Table and results are clearly presented.

2. Authors provided detailed information on patients who developed GTN, however some information limited by retrospective nature of study

3. Interval pregnancy history noted with pregnancy outcomes for those patients who developed GTN. It would be interesting to know the overall pregnancy outcomes for patients without development of GTN. Specifically, did any patients become pregnant prior to completing the recommended time for hCG surveillance and what were the outcomes?

- We thank the reviewer for highlighting this very interesting question. We do not have this data for the entire cohort included in this study, but have collected this data for a cohort from 2010-2020. Since we do not have this data available in the database for the entire cohort and we felt this subset analysis shifted the main message of the manuscript, we have not included it with this work. We plan to analyze the updated cohort with available pregnancy data and present these findings in a subsequent manuscript.

4. Table 1: Many patients' presentation at the time of GTN diagnosis was only an elevated hCG. Do you have data on why the hCG level was tested? Was it due to: missed menses? symptoms of pregnancy? Incidental testing? Often we counsel patients about symptoms of abnormal vaginal

bleeding, but knowing the context of why an hCG was collected could help with counseling patients for precautions for GTN.

- The reviewer raises an excellent clinical point here. Unfortunately, due to the retrospective design of our study, we did not have additional clinical documentation regarding the reason why the hCG was tested. We included as much information as possible from the available clinical notes, but in some cases there was limited documentation. In addition, over the 40 year time period of the study, the follow up hCG time was initially 2 years and due to evolving research follow-up was reduced as described in the methods section of the manuscript in the first paragraph on page 7. Consequently, earlier on some of the late relapses were detected as part of routine follow-up whilst more recently as the reviewer has correctly surmised they were detected for other reasons.

Discussion:

1. The data is compelling for decreasing monitoring of hCG surveillance after a complete hydatidiform mole pregnancy

2. Agree that this study can help guide shared decision making for the length of monitoring of hCG if normalization > 56 days.

Reviewer: 2

Comments to the Author
Major comments:
1. This study is a retrospective cohort study using the UK databases for molar pregnancy to evaluate the specific question of the role of ongoing hCG screening after normalization for patients with complete mole

2. The data utilized is an excellent resource for this question. The paper is generally clear, concise, and well written. The methodology appears appropriate to my level of statistical training, though I am unfamiliar with the Bayesian statistical modeling and defer to a statistical reviewer to comment on this choice
3. Generally, I do think this data likely warrants publication, but the authors need to use caution in how the frame the novelty of the data and of the conclusion. The authors state, "In keeping with prior data7,8, patients with CHM who achieved hCG normalization within 56 days had a significantly lower risk of GTN compared to those normalizing beyond this (1/1159 vs 1/308, OR 0.27 95% CI 0.08–0.88, p=0.03). Consequently, patients with CHM appear to need longer hCG monitoring if the hCG normalizes beyond 56 days from the date of uterine evacuation." This data is drawn from Coyle et al (reference 6), then just a few sentences later state "However, no data are currently available to inform us of the relative risk of malignant transformation of CHM when the hCG normalizes either within or

beyond 56 days." Coyle et al includes Charing Cross cases of CHM and PHM from 1980 to 2009, whereas the current study includes Charing Cross and Sheffield cases from 1980-2020. Therefore, my interpretation is that the current study includes the 8,400 CHM from the Coyle et al study, plus about 9000 additional cases from Sheffield or more recent years at Charing Cross. It is inaccurate to state that there are no data about relative risk with normalization within or beyond 56 days. This is reported in Coyle et al, and this topic is also assessed in the systematic review by Albright, et al, which compiles Coyle et al with data from other trophoblastic disease centers in the US (NETDC) and other countries. The conclusion of this paper is not truly novel as it is highly similar to the conclusions from both Coyle et al and Albright et al.

- Thank you for highlighting how this part of our manuscript may be confusing in light of the previous literature. We were trying to highlight that in the previous Coyle paper, the risk ratios over follow up time were not divided up by patients who normalized within 56 days or greater than 56 days. We have revised this in the manuscript to more clearly articulate the difference in our data presentation and the previously published studies.
- Deleted: "However, no data are currently available to inform us of the relative risk of malignant transformation of CHM when the hCG normalizes either within or beyond 56 days. Here, we establish these relative risks and determine whether the total duration of hCG monitoring after normalization within and beyond 56 days can be safely reduced in women who have undergone uterine evacuation of a CHM."
- Added to last sentence of introduction: "We aimed to review the relative risk of malignant transformation over time in patients with CHM who normalize within or beyond 56 days in a larger cohort of patients from 2 trophoblastic disease centres."

4. I think the detailed description of cases with GTN after hCG normalization is actually the most novel part of the study, though it is framed as a secondary aim of the analysis. I would recommend considering emphasizing this part of the analysis more in the objectives and abstract.

- Thank you for highlighting this strength of our paper. We have modified the objectives and abstract as follows to emphasize this aspect of our work.
- Abstract added:
    - **Main outcome measures** Incidence and clinical presentation of GTN after normalization of hCG following uterine evacuation of CHM.
- Manuscript methods added:
    - For patients who developed GTN, clinical presentation, interval pregnancy history was noted along with FIGO score and treatment required.

5. Related to the above point regarding data overlap, I am trying to correlate the Coyle et al data with the data in the current paper. Coyle et al reports 3 GTN cases among 3,477 CHM with normalization <56 days, and 23 GTN cases among 7,081 CHM with normalization >56 days. This paper reports 3/4804 with normalization <56 days, and 28/12,620 cases with normalization >56 days. This implies that the addition of 11 years of follow up from Charing Cross and 30 years of data from Sheffield

added no cases of GTN among ~1400 added cases with normalization <56 days, and 5 cases of GTN among ~5000 added cases with normalization >56 days. The low number of cases from the added data is somewhat surprising, but it may be accurate. I just ask that the authors re-confirm the accuracy of this data and this comparison to the Coyle et al study. I think it would be useful and informative as a sensitivity analysis to describe the data from the two centers separately for reference, as any data from Sheffield is novel and not included in the previously published Coyle et al paper. I don't have any reason to suspect differences in these populations, but it seems like the rates may have been different between the two centers potentially?

- When we reviewed the entire cohort for patients, there was 1 patient in the Coyle cohort that was reported to have normalized within 56 days, but in fact had received MTX in another country so this was not captured in the chemotherapy database and was picked up from review of the clinical notes, therefore this patient was excluded from the analysis. The remainder of cases were reviewed and correctly included.
- <56 days = 4804
    - 1980-2009 at Charing Cross: 2/3476=0.06% (1 removed as had previous chemo)
    - 2010-2020 at Charing Cross: 0/933=0% (there was 1 patient at 61 days)
    - 1980-2020 at Sheffield: 1/395=0.25%
    - Total=3/4804=0.06%
- >56 days =12620-8926
    - 1980-2009 at Charing Cross: 23/7081=0.32%
    - 2010-2020 at Charing Cross: 5/1845=0.27%
    - 1980-2020 at Sheffield: 0/3694=0%
    - Total=28/12620=0.22%

- Thank you for the suggestion of a sensitivity analysis to better understand the interaction of our variables. Given how rare the events are, this highlights the importance of a Bayesian approach, as even with data we've collected, there's still significant uncertainty which isn't captured by just looking at the numbers in isolation (i.e. without some sort of sensible prior). This will be addressed further down in response to Reviewer 3.

6. "Figure 3 shows a non-significant trend in this direction with the median time being 367 days in the single-agent group and 524 days in the multi-agent group (p=0.811)" -> a p value of 0.811 is not much of a trend, would use caution with this framing. It would be more accurate to state that there was no statistically significant difference and state medians and p value, allow the reader to interpret beyond that.

- We are grateful to the reviewer for pointing this out. We have changed the manuscript to read: 'Figure 3 shows the median time from evacuation to treatment for GTN was 367 days in the patients who achieved remission after single-agent chemotherapy compared to 524 days for

patients requiring multi-agent chemotherapy for remission.'

7. I think it would be beneficial for the discussion section to reference and help frame the data with the findings from https://www.ncbi.nlm.nih.gov/pmc/articles/PMC9941751/ (of which I am an author) which is a cost-effectiveness analysis assessing this exact question of the optimal surveillance strategy. The data is applicable to this topic/discussion.

- Thank you and we have modified the first paragraph of our discussion to reflect this:
    - 'Whilst in former times hCG monitoring after evacuation of a CHM lasted for 2 years, most current guidelines indicate that surveillance should continue for up to 6 months after normalization of this hormone. In some countries including the UK, if normalization occurs within 56 days then follow-up is reduced to 6 months from the date of evacuation.[8] As previously reported, here we show that the overall risk of developing GTN following hCG normalization is low (0.2%) with the majority diagnosed after the current 6-month hCG surveillance protocol [6, 17- 20]. Moreover, we confirmed that the risk of GTN was 3.6-fold lower in those normalizing within as opposed to beyond 56 days[6]. However, we demonstrate for the first time that the absolute risk for GTN in patients normalizing within 56 days was just 1/1601. Therefore, in patients who normalize within 56 days it is appropriate to change current UK and international practice to reduce monitoring protocols to just one confirmatory normal hCG sample to match current practice for PHMs[9]. Interestingly, a recent Markov model-based cost-effectiveness analysis in CHM also suggested that it would be reasonable to reduce or potentially stop hCG monitoring after hCG normalization[21]. '

8. I agree with the author's conclusions that the surveillance guidelines for complete mole could likely be further adjusted based on this and related data. I do think it would be helpful to frame this recommendation by describing in more detail how recommendations for surveillance have already evolved in recent years from the traditional 6 months or more of follow up for partial and complete mole to the current guidelines by RCOG and FIGO/ACOG.

- We agree with the reviewer and have rewritten the first paragraph of the discussion:
    - 'Whilst in former times hCG monitoring after evacuation of a CHM lasted for 2 years, most current guidelines indicate that surveillance should continue for up to 6 months after normalization of this hormone. In some countries including the UK, if normalization occurs within 56 days then follow-up is reduced to 6 months from the date of evacuation.[8']

Minor comments:

1. Many of the references in the reference list are duplicated and given 2 different numbers (6=26, 7-27, 18=36 for example).

- Thank you for identifying this, all duplicates have been removed.

2. In the introduction, when describing the typical course of molar pregnancy, I think it is worth mentioning that the natural course of a molar pregnancy is likely to result in spontaneous abortion and some molar pregnancies may be unrecognized early spontaneous abortions.

- We have modified the introduction as follows:
    - 'Some molar pregnancies may be undetected as early spontaneous abortions, but when an abnormal pregnancy suggestive of a molar pregnancy is detected on an early pelvic ultrasound this results in a suction uterine evacuation.'

3. "In contrast, for CHM we noted a slightly higher risk of post-molar GTN after hCG normalization (0.24%, n=20/8400) which decreased from 1/839 at 4 months to 1/1677 patients at 12 months after the 1st normal hCG value6. -> would be helpful to put the percentages rather than fractions for easier comparisons between the values at the different time points. This same point about putting percentages in addition to fractions applies to the results section as well.

- We have provided the percentage in brackets in the introduction and results as recommended by the reviewer. We have left the fractions as well as we think it is important to highlight the denominator of patients in the study population when assessing rare outcomes.

Reviewer: 3

Comments to the Author

Thank you for the opportunity to review this interesting paper. It is clear the authors have worked hard to produce this research, examining a rare event from 40 years of data. I have some comments from a statistical point of view.

1) Abstract says: 'The risk of developing GTN following uterine evacuation' – here and in the whole article, I am looking for the time-point of interest. Risk by when, exactly?

- We explain the background to the chosen time window in more detail in response to points 3-7. The time point of interest is the number of days between uterine evacuation to hCG normalization and to diagnosis of GTN. As the diagnosis of GTN is difficult to measure retrospectively, we used the start of GTN treatment as the time point, as treatment is started almost always within 24 h after diagnosis. The latest diagnosis of GTN (in other words, start of GTN treatment) was 2697 days after hCG normalization.

2) Mantel Haenzel tests and relative risks are derived, but these approaches assume (I think) the data are binary outcomes without censoring before the time-point of interest for the event risk to be defined by. But surely there is censoring due to loss to follow-up (i.e. different lengths of follow-up need to be accounted for) over time, such that not all individuals event status is known by the time point of interest?

- Thank you for your comment. We have chosen to continue with the Bayesian approach and remove the Mantel Haenzel test. See the points below for further explanation.

3) A related point is that equations 1 to 3 shown a 'time to event' model but this is not actually accounting for censoring (which the name 'time to event' model might usually imply) but simply is a logistic regression model with an interaction with time.

- The model specified here is a Bayesian version of the traditional Kaplan-Meier estimator. The maximum likelihood Kaplan-Meier estimator of the continuous time-event survival function can be defined as $S(t) = \prod_{i:t_i<t} \frac{n_i - d_i}{n_i}$. The essence of this model is therefore a conditional probability of an event occurring within a time interval j, defined as the proportion of the events that occur within time interval j, out of all the possible individuals that are at risk at the beginning time interval j: $p(t_j) = \frac{n\,events_j}{n\,atrisk_j}$ This conditional probability can therefore be appropriately modelled with a binomial (that decomposes to a Bernoulli given that it's a binary outcome) likelihood to estimate $p(t_j)$, as specified in the text, within each time interval that is generated when an event occurs, as per a traditional Kaplan-Meier analysis. Given that the model uses the number of patients at risk to be those that *remain* following removal of those that have already experienced the event, the analysis is right-censored to those that have developed disease. The table below shows the aggregated data, discretized into time intervals, as per a traditional Kaplan-Meier analysis, that are used as the input for the model. The model uses these data (and prior) to estimate $p(t_{ij})$ within each time interval j, within each patient group i. We have included the traditional Kaplan-Meier estimates in the table below for reference (produced using the {*survival*} R package). See here for more information on Bayesian formulations of these models.

Patient group (hCG normalisation)	Time interval	Time of event occurrence (months)	n_{risk}	n_{events}	Naive $p(t_{ij})$ estimate (i.e. traditional KM estimate)	Bayesian model $p(t_{ij})$ posterior median
< 56 days	1	3	4804	1	0.00021	0.00009
< 56 days	2	11	4803	1	0.00021	0.00009
< 56 days	3	39	4802	1	0.00021	0.00009
≥ 56 days	1	0	12620	1	0.00008	0.00004
≥ 56 days	2	1	12619	2	0.00016	0.00011

≥ 56 days	3	2	12617	3	0.00024	0.00018
≥ 56 days	4	4	12614	2	0.00016	0.00011
.≥ 56 days	5	6	12612	1	0.00008	0.00004
≥ 56 days	6	7	12611	1	0.00008	0.00004
≥ 56 days	7	8	12610	1	0.00008	0.00004
≥ 56 days	8	9	12609	2	0.00016	0.00011
≥ 56 days	9	12	12607	2	0.00016	0.00011
≥ 56 days	10	14	12605	1	0.00008	0.00004
≥ 56 days	11	16	12604	2	0.00016	0.00011
≥ 56 days	12	22	12602	1	0.00008	0.00004
≥ 56 days	13	24	12601	1	0.00008	0.00004
≥ 56 days	14	28	12600	2	0.00016	0.00011
≥ 56 days	15	31	12598	1	0.00008	0.00004
≥ 56 days	16	35	12597	1	0.00008	0.00004
≥ 56 days	17	43	12596	1	0.00008	0.00004
≥ 56 days	18	58	12595	1	0.00008	0.00004
≥ 56 days	19	68	12594	1	0.00008	0.00004
≥ 56 days	20	88	12593	1	0.00008	0.00004

4) I am confused as to why time is categorised. Surely a more complete picture is to model the log-odds of event as a function of time, allowing for non-linear relationship with say splines?

- The 56 days cut-off has been used by previous studies (Coyle et al., Bagshawe et al.,1986). Bagshawe et al., 1986 was the first study to propose the 56 day cut-off as they found that patients who normalized within 56 days did not require any chemotherapy (42% of 8095 patients registered). Since then, the division into these 2 groups based on the number of days between the uterine evacuation and the hCG normalization has remained an important clinical factor.

*5) I have no issue with taking a Bayesian approach, or the use of informative prior distributions, but here I did wonder whether the prior distribution is dominating the data given how sparse the outcome is. The prior given seems to be specified on the log odds of the event being low, but I cannot see what the prior is on the actual effect (Relative risk) of interest, or how informative this might be. Indeed, in equation (2), it looks like this is avoided by modelling the log-odds for each group*time separately, and with the prior the same on the log-odds for each; thus am I right that this is equivalent to*

assuming no effect (difference) between groups a priori? Post-estimation, I assume a function of the alpha_ij terms are then used to make inferences about differences in effect?

- With reference to the answer to point 5, the reviewer is correct that the log-odds for each time interval and patient group are estimated separately, as per a traditional Kaplan-Meier analysis (you can convert the probabilities to log-odds, as required). As the same prior is used for each time/group combination, prior to seeing the data, the model conservatively assumes that there is no difference between the patient groups. The reviewer is correct that we can then make inferences *between* patient groups by using the relevant posterior distributions. The data are indeed sparse, and that was the rationale behind using a Bayesian approach, as traditional maximum likelihood/ordinary least-squares methods often provide biased parameter estimates in the context of rare events. Our rationale was also such that *a priori*, we assumed that the rate of GTN formation would be very low in both groups, but were willing to let the data change our minds if there was enough evidence. As can be observed in the Table within the response to point 5, where the data are particularly sparse, the Bayesian model pulls the inference towards the prior. Moreover, the posterior estimates better capture our prior uncertainty about the risk of events occurring. We did also fit a zero-inflated binomial model to the data, given the sparsity of the outcome; however, we did not see a substantial difference between the models (as assessed by leave-one-out cross-validation) - the estimates of this model are also shown in Figure R1 for comparison (alongside a traditional Kaplan-Meier analysis).

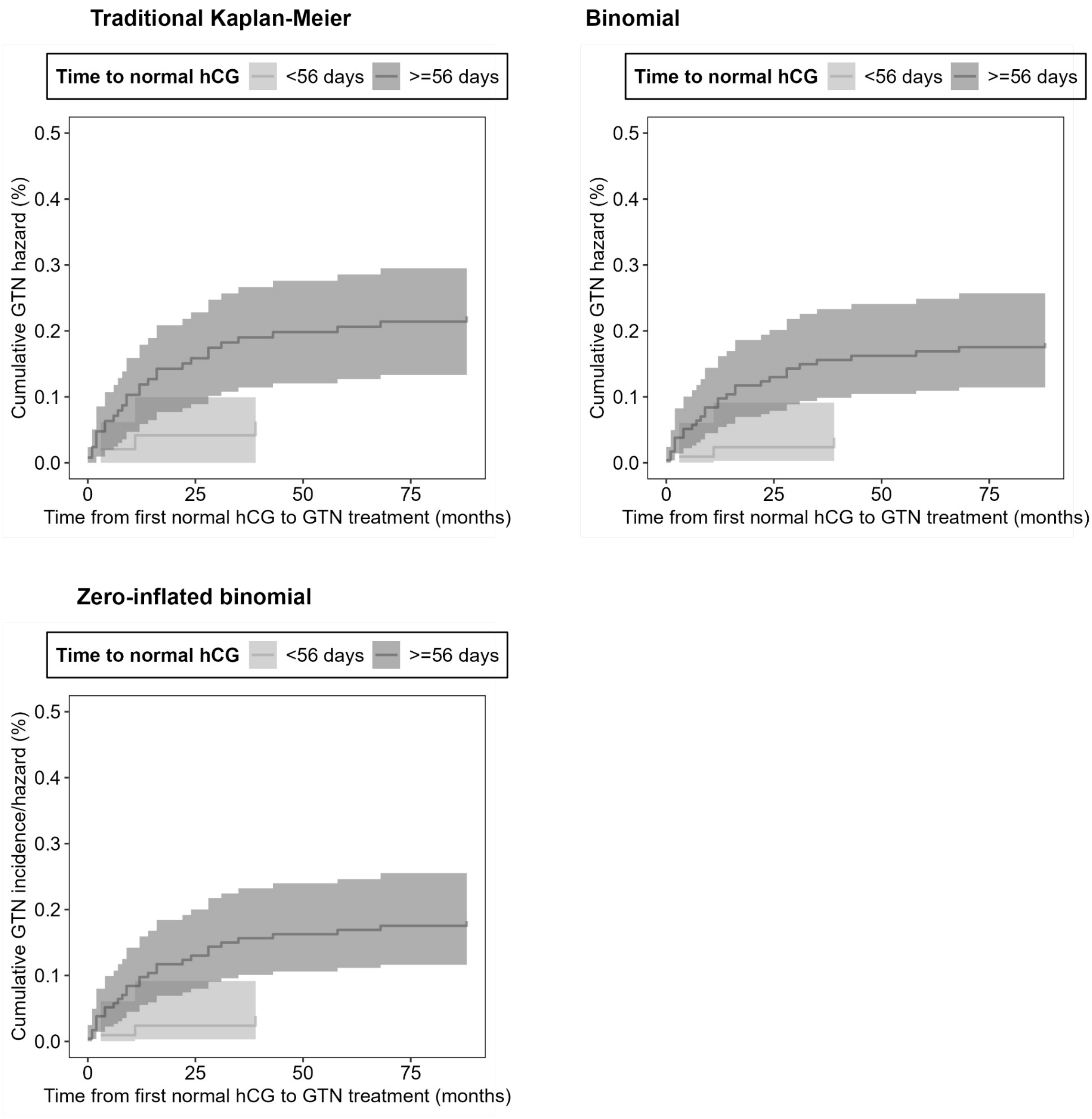

Figure R1. A comparison of different modelling approaches. Top-left, a traditional Kaplan-Meier model. Top-right, a Bayesian survival Bayesian model as specified in the text. Bottom-left a Bayesian zero-inflated binomial fitted to the data. Shaded areas represent 95% confidence (Kaplan-Meier) or credible (Bayesian) intervals. Solid lines represent the point estimate (Kaplan-Meier) or median of the posterior (Bayesian) in each case.

We also performed a sensitivity analysis to determine the influence of the prior on the results and used a less informative prior than our original analysis in each case (increasing the prior SD from 2 to 3). Figures R2-R4 show a comparison of five different priors with different location parameters on the log-odds scale. Figure R2 shows the densities of the prior (grey) and posterior (coloured according to patient group) distributions. As can be observed, the posterior distributions remain relatively invariant to the position of the prior. Figure R3 demonstrates these same models' results on the probability scale, as survival curves. Figure R4 demonstrates more clearly that the choice of prior does not

influence the inference that the rates of GTN incidence are different between the two patient groups. The R code used to reproduce all of these analyses is provided in the supplementary material.

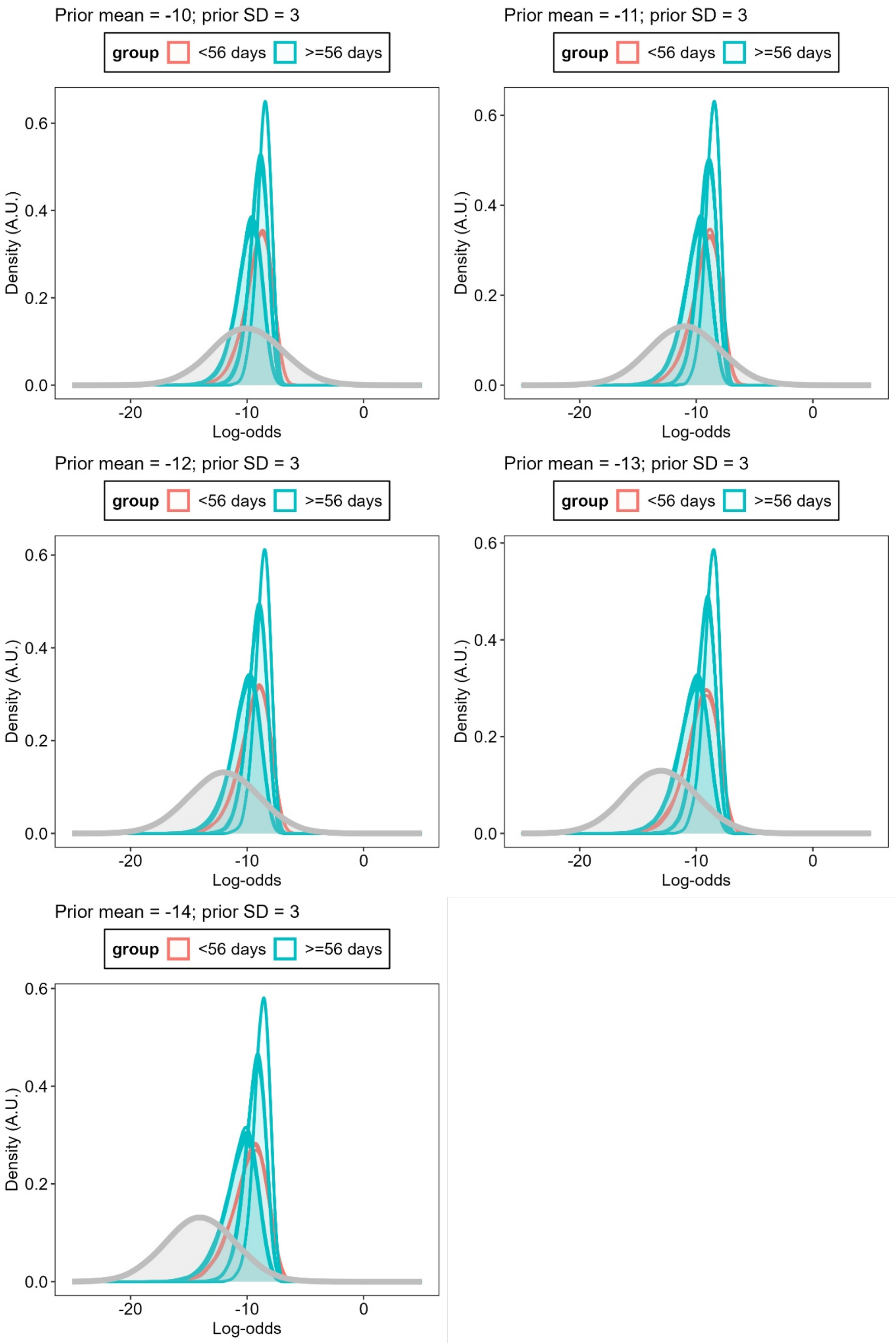

Figure R2. A comparison of priors for the Bayesian survival model - posterior distributions on the log-odds scale. Prior distributions are shown in grey. Posterior distributions are colored according to patient group.

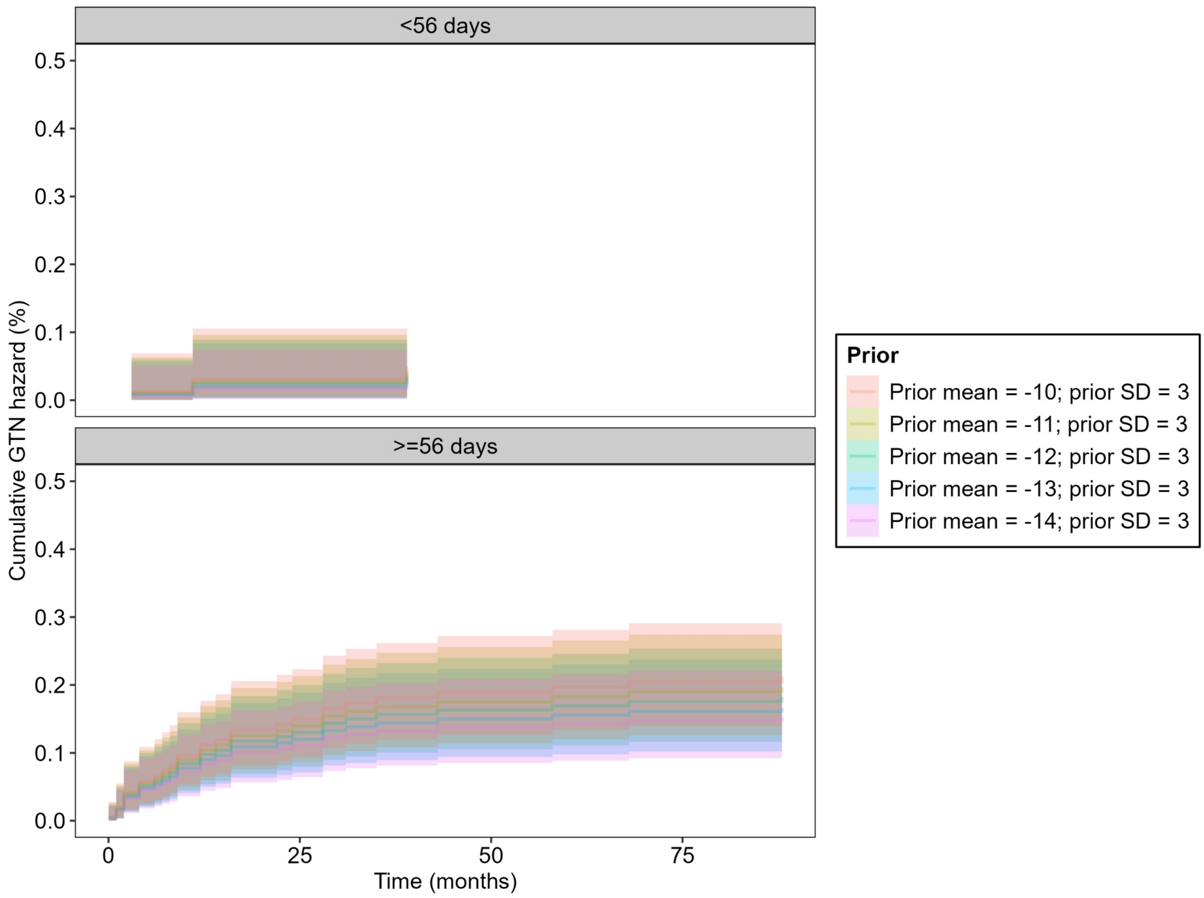

Figure R3. A comparison of priors for the Bayesian survival model - posterior distributions as survival curves. Shaded areas represent 95% credible intervals. Solid lines represent the median of the posterior in each case.

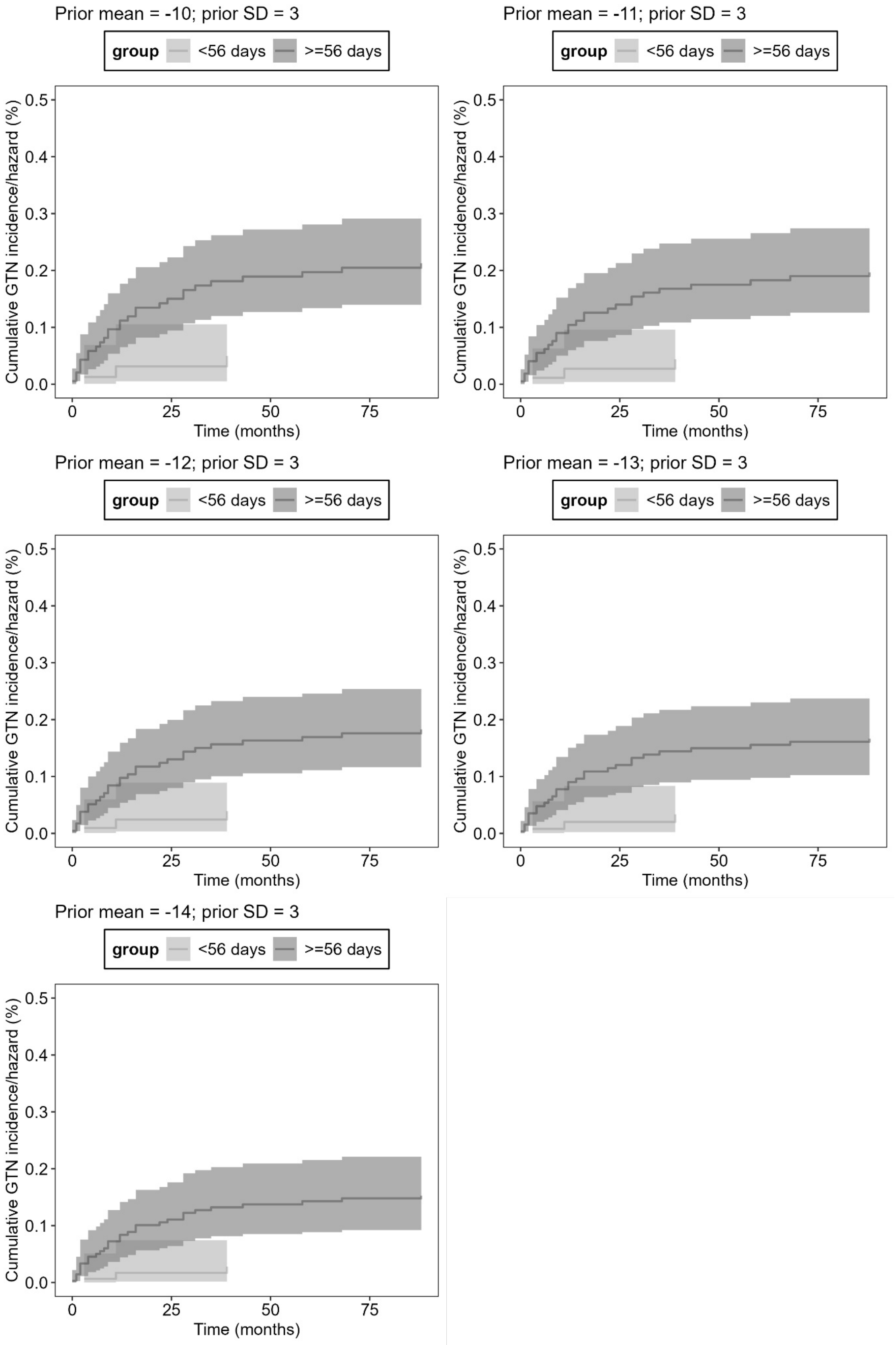

Figure R4. A comparison of priors for the Bayesian survival model - posterior distributions as survival curves. Shaded areas represent 95% credible intervals. Solid lines represent the median of the posterior in each case.

6) The results are given as risks and relative risks, and yet the actual Bayesian model is specified in terms of odds

- The Bayesian models are specified on the log-odds scale for ease of computation as per an equivalent standard general linear model; however, the outputs can easily be converted to the probability scale with the inverse logit function (as is presented in Fig 2 of the manuscript and Figs R1 and R3-R4).

7) Is it time after the test result? Or the time after the uterine evacuation that is being modelled?

- Our outcome measure is the time from the moment of uterine evacuation to hCG normalization for the division of the 2 groups and then the time from uterine evacuation to the event of GTN diagnosis (measured by hCG elevation).

8) If GTN occurred before the test result – but then later there was a normalization test result – how was this handled in the analysis? The patients were excluded I think, so the results only apply to those for whom GTN had not occurred before hand. This needs to be made clearer. A broader question is whether we have a somewhat selective group of individuals who can end up with a normal test result (perhaps that does not even matter). For example, one person died of a different (?) issue – who were they handled in the analysis?

- We excluded all patients who developed GTN before hCG normalization, as these patients are treated with chemotherapy immediately after GTN diagnosis. They have a different risk profile than patients who develop GTN after hCG normalization.

9) P-values are provided and yet a Bayesian approach is taken, which are not compatible.

- While we might disagree that frequentist and Bayesian are completely incompatible (see Gelman and Henning, 2017), we have replaced the traditional null hypothesis significance testing in the manuscript with an equivalent Bayesian model as specified in the text. These can also be fully reproduced in the R code available in the supplementary material.

10) Given that the study spans 40 years, I did expect to see some examination of the impact of years from 1980 to 2020. Could there be confounding by year? For example, if testing is delayed since 2000 (so more normal tests post 56 days), and the incidence of the outcome is gradually growing over time too.

- We chose this cohort, consisting of a large period of time, because of the low incidence of cases. This indeed means that we also chose for the corresponding risk of heterogeneity in diagnostics. In the discussion we mention the limitations: monitoring after hCG normalization

has changed over the years from 2 years to 6 months. We have also added the following sentence about possible limitations of follow-up in recent cases:

- Another limitation in the recent cases of GTN may have been the follow-up time. The most recent cases were diagnosed with CHM pregnancy in November 2020, resulting in a follow-up of 2 years. This could have underestimated the number of GTN diagnoses, as 12/31 patients were diagnosed with GTN 24 months after hCG normalization.

11) Relative risks and CIs should always be given for each comparison (in addition to the risks by each group separately)

- The risks/hazards have been updated with the relevant 95% credible intervals as derived from the model.

12) 'Figure 3 shows a non significant trend in this direction with the median time being 367 days in the single-agent group and 524 days in the multi-agent group (p=0.811).' – this seems a strong emphasis without any statistical justification. Just say further research is needed on this.

- Thank you for this point, we have revised this in the manuscript results:
    - Figure 3 shows the median time from evacuation to treatment for GTN was 367 days in the patients who achieved remission after single-agent chemotherapy compared to 524 days for patients requiring multi-agent chemotherapy for remission (p=0.811).

13) Another broad question is why the emphasis on 56 days? Why not 28 days or 74 days? It would have been helpful to examine this without categorisation, to see if 56 days is truly the best place for this subjective cut-off.

- 56 days was selected due to previous work showing a lower risk of GTN when hCG normalization occurs within 56 days. This previous work has informed the current duration of hCG surveillance guidelines for CHM pregnancies, so we used this as a clinically significant time point.

In summary, there are a number of areas for revision and clarification, before BMJ Medicine can evaluate this further. Some of queries may reflect a lack of clinical understanding of this particular area, but generally I hope my comments are constructive for the authors moving forwards.

VERSION 2 – REVIEW

**REVIEWER NAME**	Riley, Richard
**REVIEWER AFFILIATION**	University of Birmingham, Institute of Applied Health Research
**REVIEWER CONFLICT OF INTEREST**	None
**DATE REVIEW RETURNED**	09-Dec-2024

**GENERAL COMMENTS**	I thank the authors for their responses, and in particular the detailed explanation of the Bayesian version of the KM approach, which was helpful for me to understand the modelling behind the scenes, which I do think is appropriate. Indeed, I think in sparse data situations it really makes sense to use a Bayesian approach and to have conservative priors, so that any extreme effects are pulled toward the prior (null), which is the case here. It is a very sensible analysis framework. The paper is also very well written. I have just a few remaining comments<br><br>1) I am still surprised to see that the authors continue to only use the 56 day cut-point, and to not consider modelling this on the continuous scale, stating in their response that 'The 56 days cut-off has been used by previous studies …. since then, the division into these 2 groups based on the number of days between the uterine evacuation and the hCG normalization has remained an important clinical factor.' – even though it has become the standard to dichotomise, and thus I agree with this being an important focus, I still do not understand the (biological) rationale for considering two people as different if they are at 55.9 and 56.1 days, and two people the same if they are at 1 and 55 days. It seems a missed opportunity to apply more comprehensive and powerful modelling on the continuous scale. I recommend that, at the very least, some discussion about this is needed in the Discussion.<br>2) I like the response showing the impact of different choices of prior distributions (and Figure R2 and R3, which are superb), and these are not included in the supp material … but I could not find any text in the paper that actually discussed the impact of the choice of prior distribution or that discussed these figures. Did I miss it? If not, please add some discussion for the reader about these figures, in the main paper.<br>3) I am still confused by the outcome timing for the 'risk' statements. The authors have not addressed this. For example, the authors say "The risk of developing GTN following uterine evacuation was significantly lower if hCG normalized within 56 days compared to normalization beyond 56 days (posterior medians 0.06% [95% credible interval 0.01%, 0.14%] vs 0.22% [95% credible interval 0.15%, 0.31%]), with a posterior relative risk of 0.25 (95% credible interval 0.06, 0.72)" – but risk by when exactly? As this is a time-to-event analysis, the risk needs to come with a time-point, as otherwise the risk (and potentially relative risk) changes over time. Please address. Is it lifetime risk? 3 months risk? 9 months risk? I can't tell. If the authors want a measure of relative risk that corresponds to averaging across the whole follow-up, then a hazard ratio would be more apt.<br>4) Equations (1) to (6): underneath please define 'interval_i' and 'group_j' (the coding I mean)<br>5) 'Strikingly, the majority of patients (71.0%, n=22/31) were diagnosed beyond the existing hCG surveillance protocol.' – is this protocol 6 months? Please add the time period of the existing protocol.<br>6) Under Figure 2 it refers to 'relapse hazard' but I think it should say 'relapse risk'?<br>7) Figure 3: again 'hazard curves' should be 'recurrent risk curves'? Or cumulative incidence curves?<br>8) Figure 2: labels need to be added to tell the reader what the posterior and prior distributions actually are. What's the grey distribution and why are there 3 distributions for the >56 day group?

VERSION 2 – AUTHOR RESPONSE

Reviewer: 3

Comments to the Author
I thank the authors for their responses, and in particular the detailed explanation of the Bayesian version of the KM approach, which was helpful for me to understand the modelling behind the scenes, which I do think is appropriate. Indeed, I think in sparse data situations it really makes sense to use a Bayesian approach and to have conservative priors, so that any extreme effects are pulled toward the prior (null), which is the case here. It is a very sensible analysis framework. The paper is also very well written. I have just a few remaining comments

1) I am still surprised to see that the authors continue to only use the 56 day cut-point, and to not consider modelling this on the continuous scale, stating in their response that 'The 56 days cut-off has been used by previous studies …. since then, the division into these 2 groups based on the number of days between the uterine evacuation and the hCG normalization has remained an important clinical factor.' – even though it has become the standard to dichotomise, and thus I agree with this being an important focus, I still do not understand the (biological) rationale for considering two people as different if they are at 55.9 and 56.1 days, and two people the same if they are at 1 and 55 days. It seems a missed opportunity to apply more comprehensive and powerful modelling on the continuous scale. I recommend that, at the very least, some discussion about this is needed in the Discussion.

- We would like to thank Professor Riley for his statistical experience and helpful comments to consider for improving our work. To address the suggestion above we have added the following to the limitations section of our discussion.
    - In our analysis we used the clinically relevant cut off time of 56 days for hCG normalization as these patients underwent different lengths of hCG surveillance due to previously identified risk for developing GTN[7,8]. Future work could consider time to hCG normalization as a continuous variable in the model to more rigorously elucidate the importance of time to hCG normalization and risk of developing GTN.

2) I like the response showing the impact of different choices of prior distributions (and Figure R2 and R3, which are superb), and these are not included in the supp material … but I could not find any text in the paper that actually discussed the impact of the choice of prior distribution or that discussed these figures. Did I miss it? If not, please add some discussion for the reader about these figures, in the main paper.

- We have clarified this by adding the following sentence to the method section:
    - More detailed information regarding a comparison of the statistical models used here and an analysis of the influence of the chosen prior distributions is shown in supplementary figures 2-4. The latter analysis demonstrates how, whilst the prior regularizes the model's inferences, the data have a strong influence on the posterior.

3) I am still confused by the outcome timing for the 'risk' statements. The authors have not addressed this. For example, the authors say "The risk of developing GTN following uterine evacuation was significantly lower if hCG normalized within 56 days compared to normalization beyond 56 days (posterior medians 0.06% [95% credible interval 0.01%, 0.14%] vs 0.22% [95% credible interval 0.15%, 0.31%]), with a posterior relative risk of 0.25 (95% credible interval 0.06, 0.72)" – but risk by when exactly? As this is a time-to-event analysis, the risk needs to come with a time-point, as otherwise the risk (and potentially relative risk) changes over time. Please address. Is it lifetime risk?

3 months risk? 9 months risk? I can't tell. If the authors want a measure of relative risk that corresponds to averaging across the whole follow-up, then a hazard ratio would be more apt

- For clarification, we have qualified the overall risk of developing GTN during the entire follow up period.

4) Equations (1) to (6): underneath please define 'interval_i' and 'group_j' (the coding I mean)

- We have defined the terms under the equations.

5) 'Strikingly, the majority of patients (71.0%, n=22/31) were diagnosed beyond the existing hCG surveillance protocol.' – is this protocol 6 months? Please add the time period of the existing protocol.

- The first paragraph in the discussion describes the standard follow up during the study period:
    - Whilst in former times hCG monitoring after evacuation of a CHM lasted for 2 years, most current guidelines (American College of Obstetricians and Gynecologists, Royal College of Obstetricians and Gynaecologists, International Federation of Gynaecology and Obstetrics) indicate that surveillance should continue for up to 6 months after normalization of this hormone[8]. In some countries including the UK, if normalization occurs within 56 days then follow-up is reduced to 6 months from the date of evacuation[8].

6) Under Figure 2 it refers to 'relapse hazard' but I think it should say 'relapse risk'?

- Thank you for your suggestion. The hazard function is what we're estimating with the model (or traditional Kaplan-Meier) and is the opposite of the survival function. However, we understand the reviewer's point and have changed "hazard" to "risk" to make it clearer. We have changed the title and the text of figure 2 and table 1 to:
    - Title: Relapse risk curves of GTN development over time after hCG normalization - additional relapse risks can be found in table 1.

7) Figure 3: again 'hazard curves' should be 'recurrent risk curves'? Or cumulative incidence curves?

- Thank you for your suggestion, we have changed "hazard curves" to "cumulative incidence curves".

8) Figure 2: labels need to be added to tell the reader what the posterior and prior distributions actually are. What's the grey distribution and why are there 3 distributions for the >56 day group?

We've updated these in the attached supplementary figures according to Prof. Riley's suggestions. The three distributions are due to the similarity of the hazard function estimates over several different time points (which are included in the table under point 3 in the responses to Prof Riley).
